# Supplementary material for: New Organoselenium (NSAIDs-Selenourea and Isoselenocyanate) Derivatives as Potential Antiproliferative Agents: Synthesis, Biological Evaluation and in Silico Calculations
Source: Molecules. 2022 Jul 6;27(14):4328. doi: 10.3390/molecules27144328 (PMC9320890; doi:10.3390/molecules27144328)
Supplement: Supplementary file 1 [file molecules-27-04328-s001.zip › molecules-1752243-supplementary.pdf]

Supporting information

Table of Contents:

Content of supporting information

	Content	Page
2	Copies of ^{1}H, ^{13}C NMR and MS spectra of compounds	S2

4a ^{1}H NMR

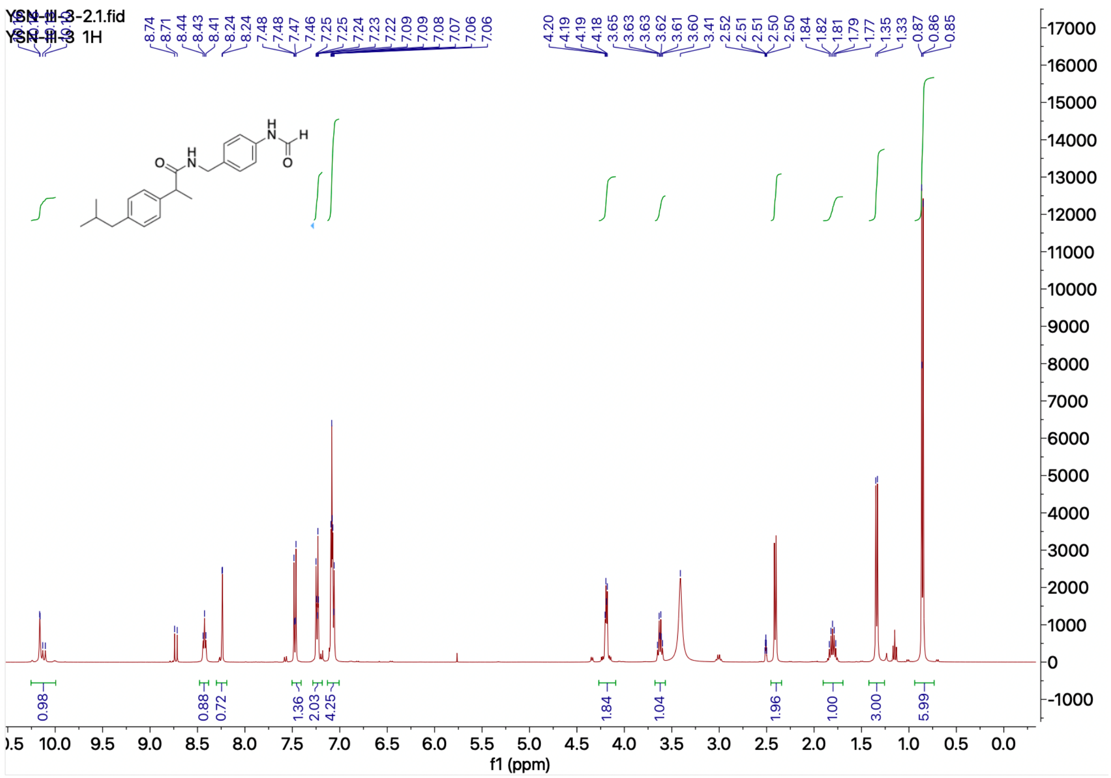

4a ^{13}C NMR

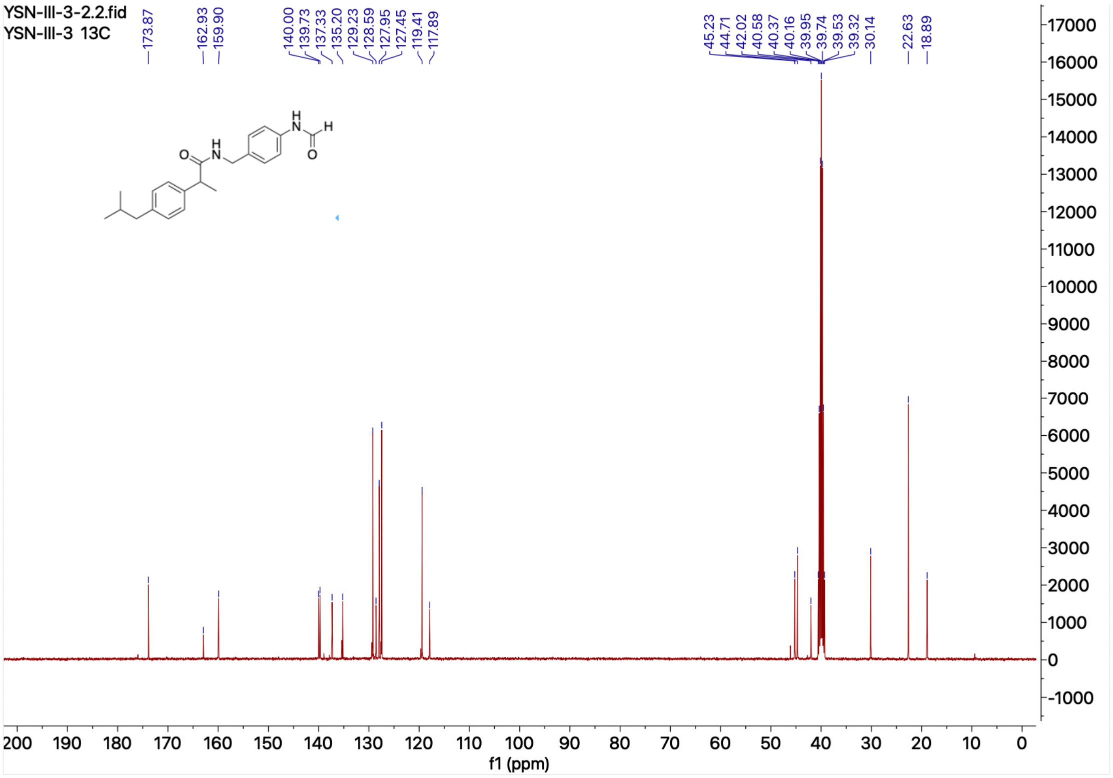

4b ^{1}H NMR

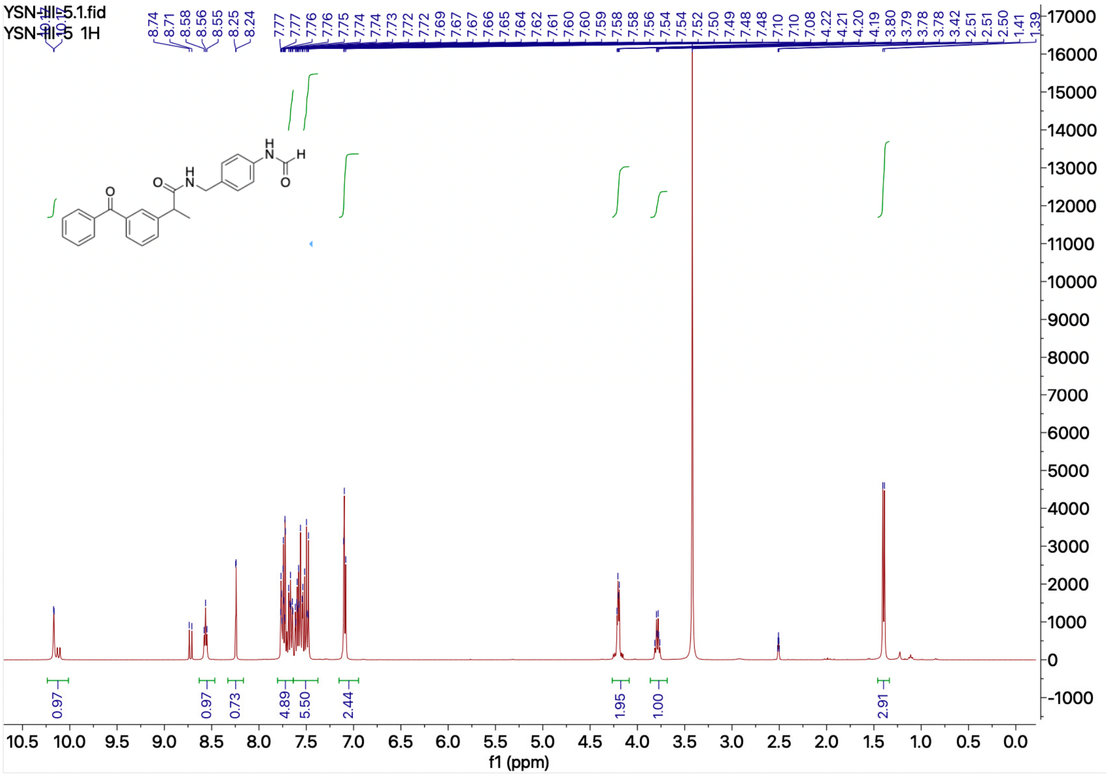

4b ^{13}C NMR

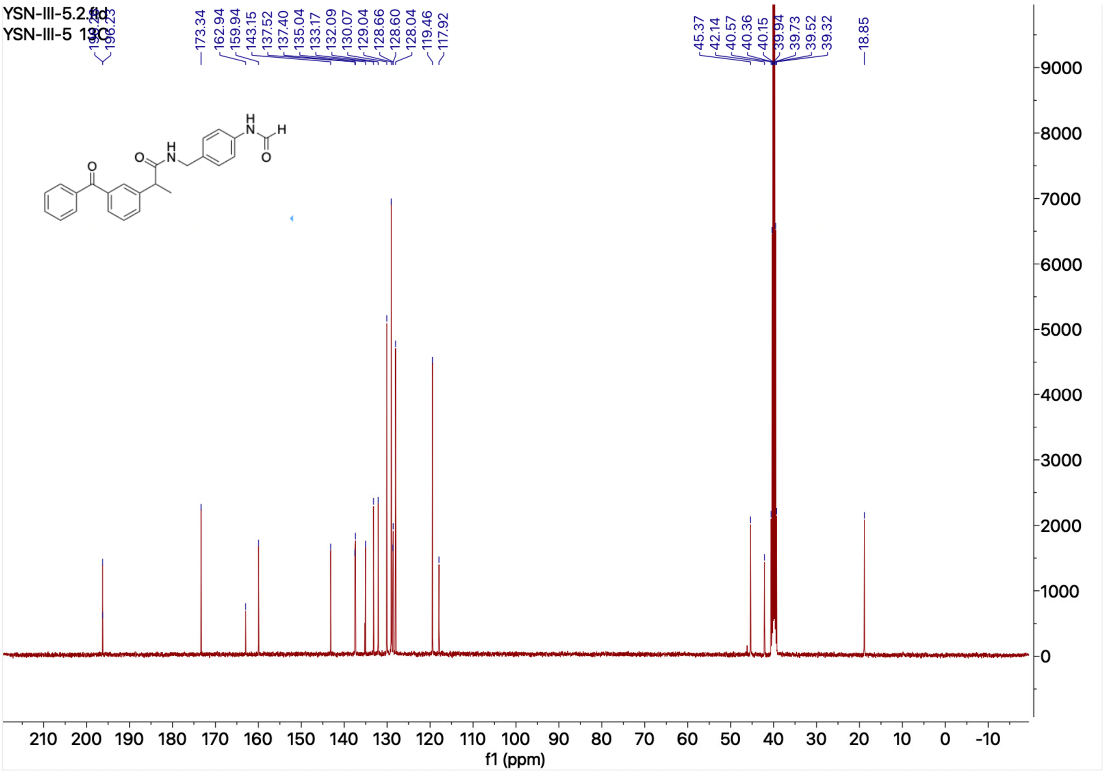

4c ¹H NMR

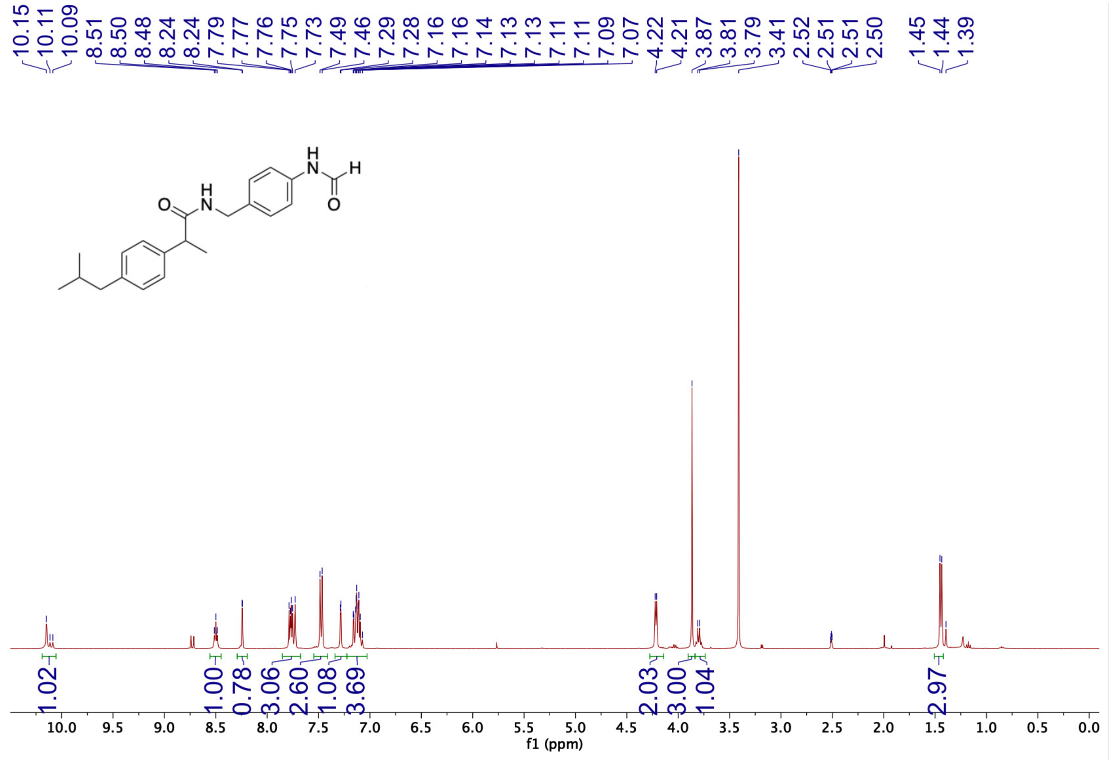

4c ¹³C NMR

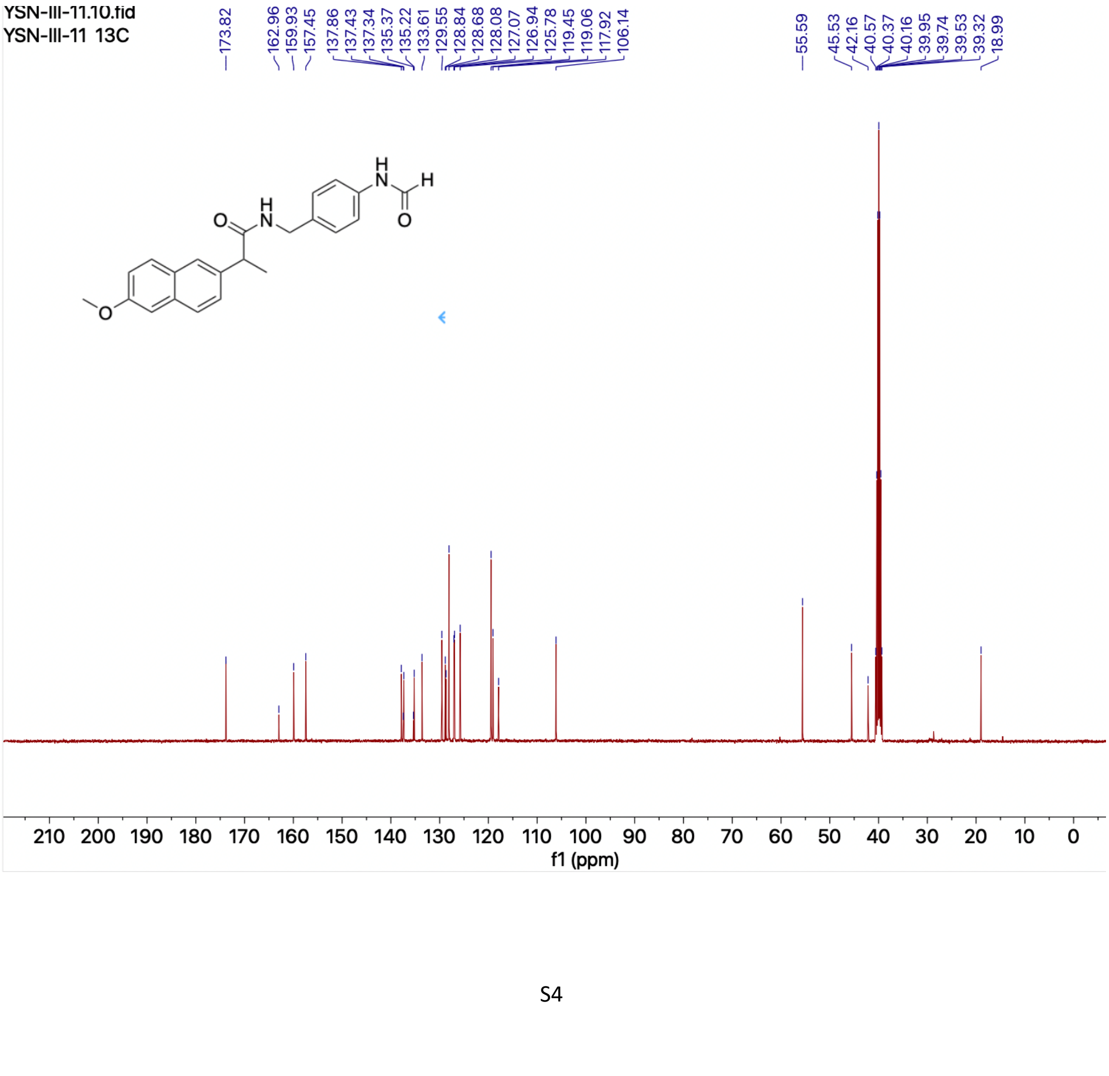

4d ¹H NMR

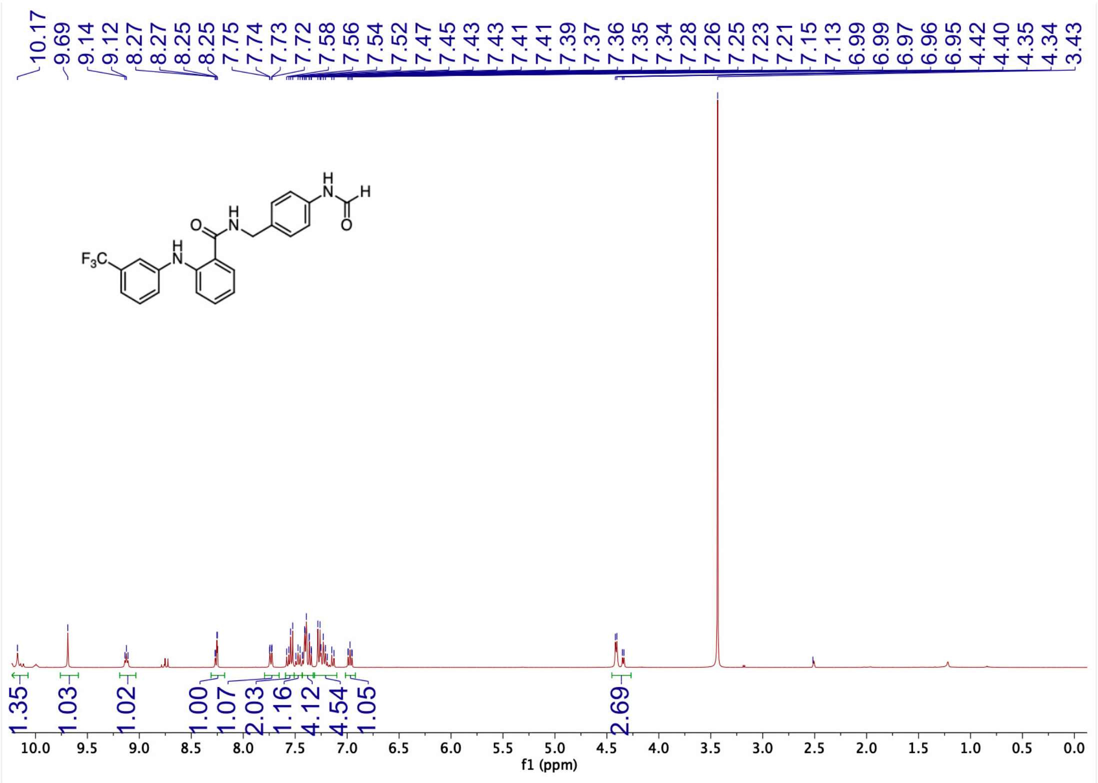

4d ¹³C NMR

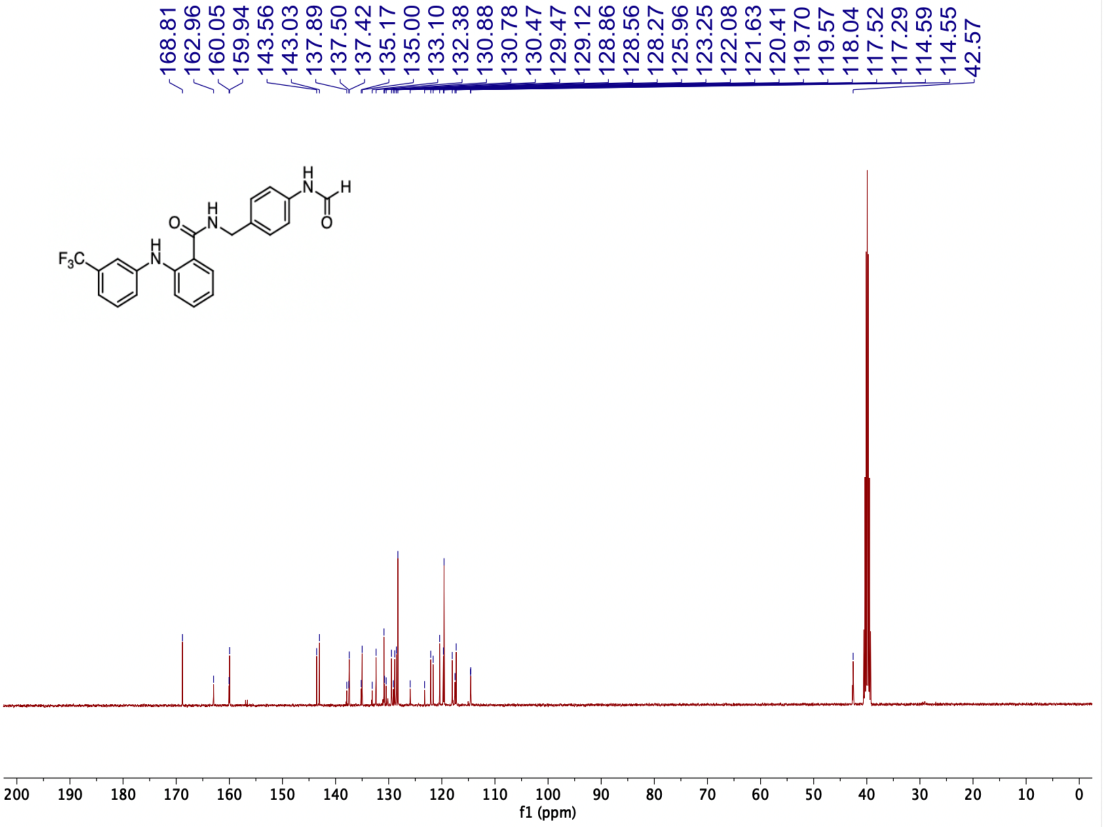

4e ¹H NMR

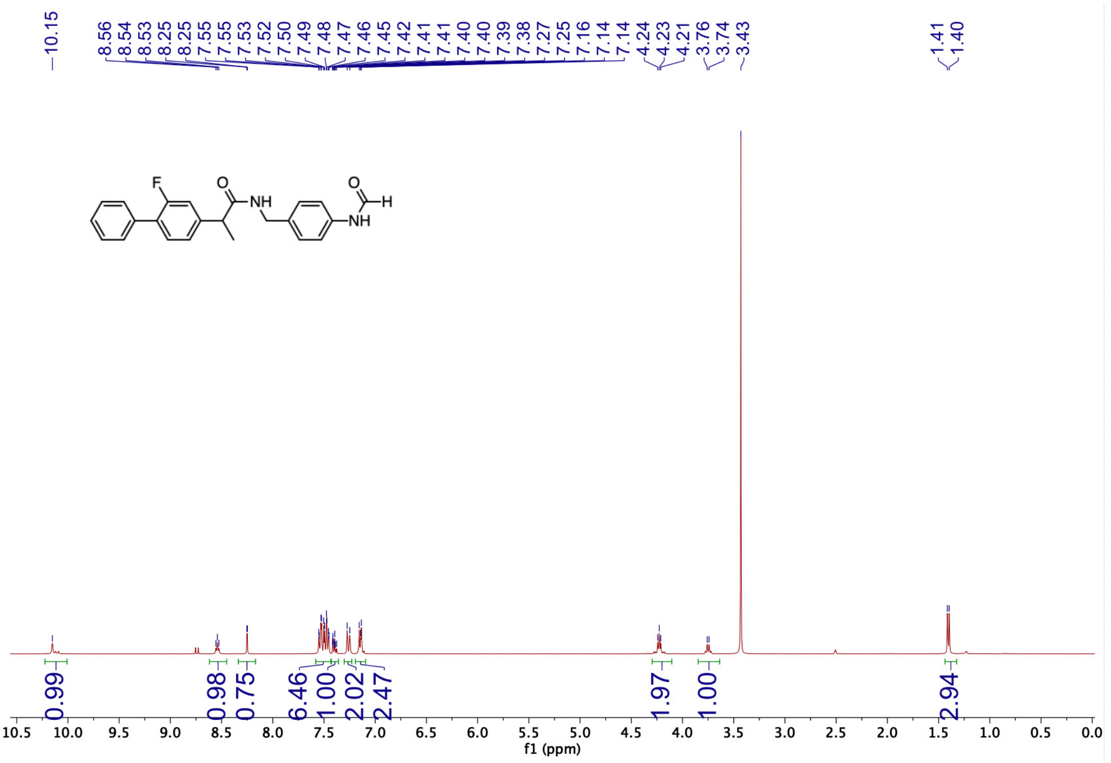

4e ¹³C NMR

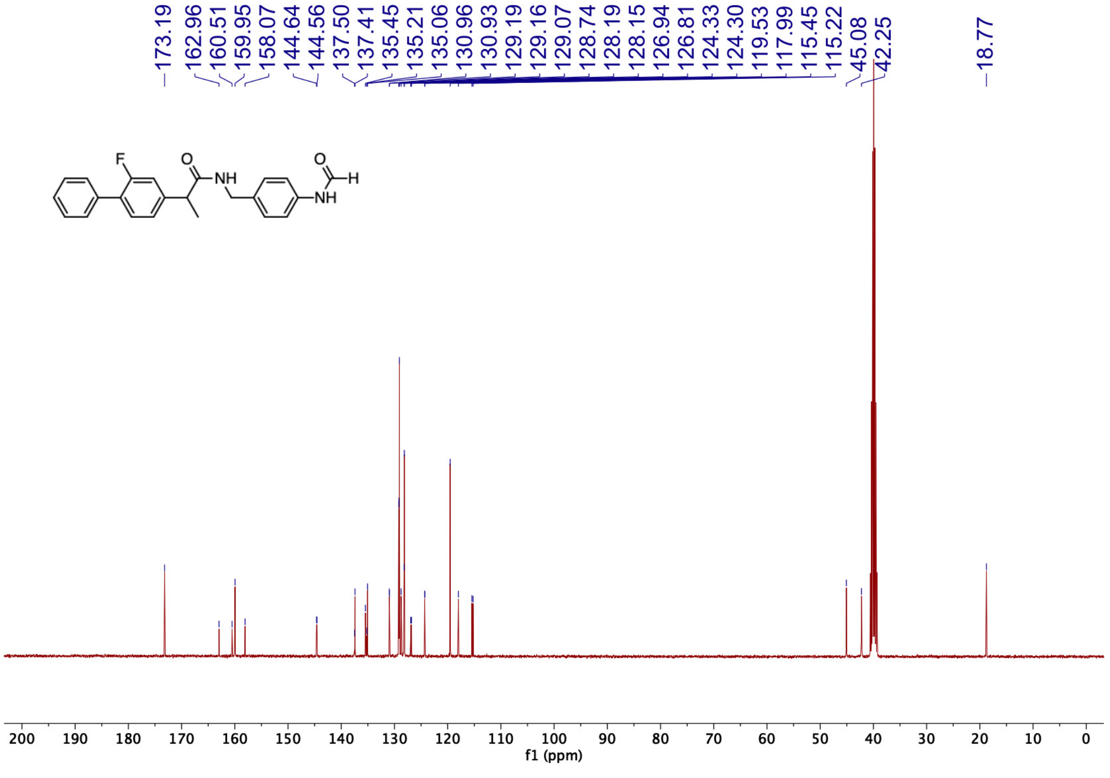

4f ^{1}H NMR

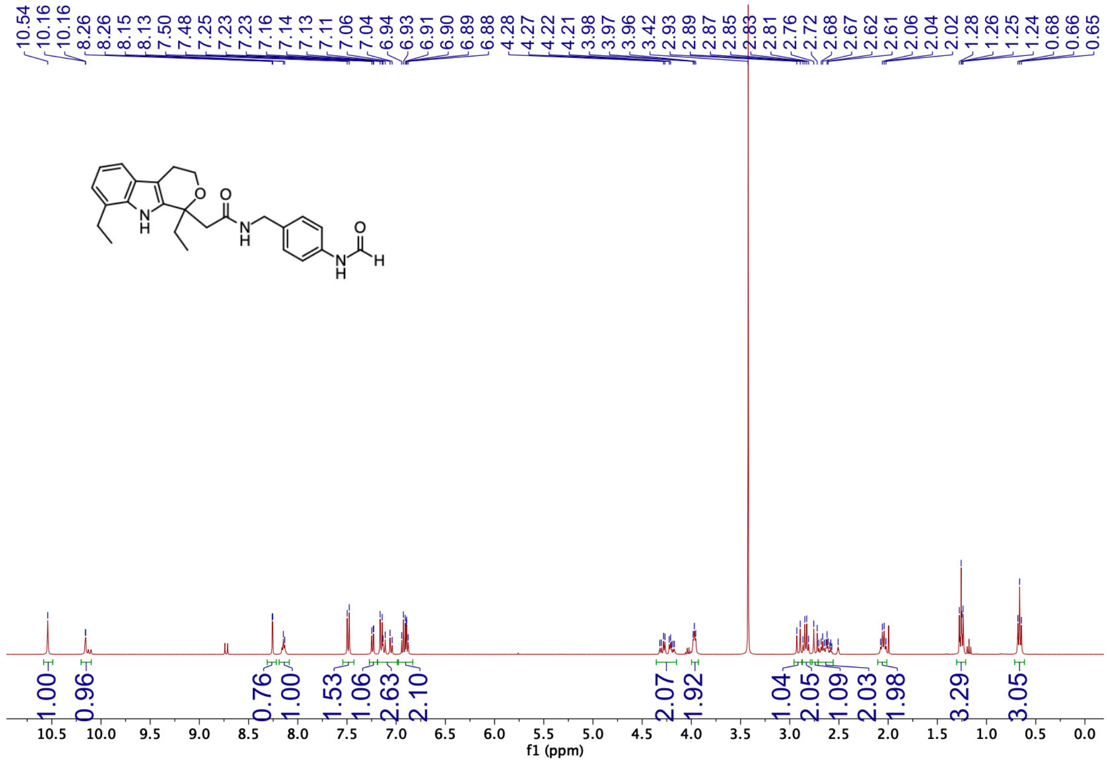

4f ^{13}C NMR

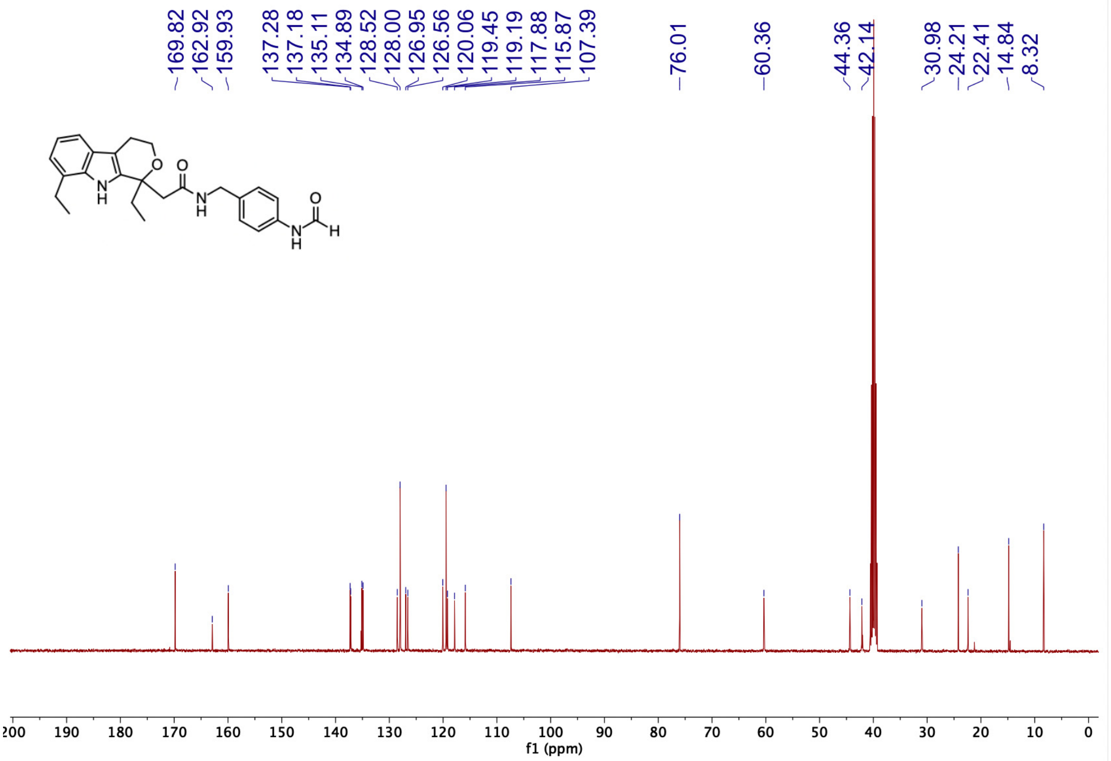

4g ^{1}H NMR

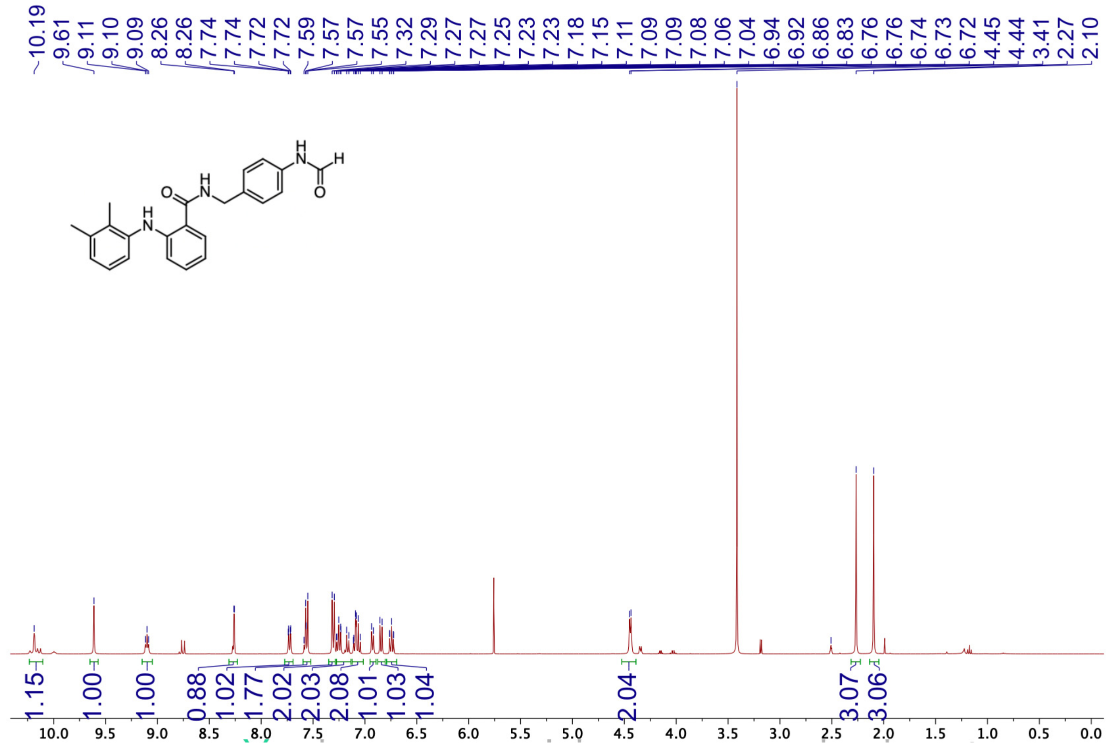

4g ^{13}C NMR

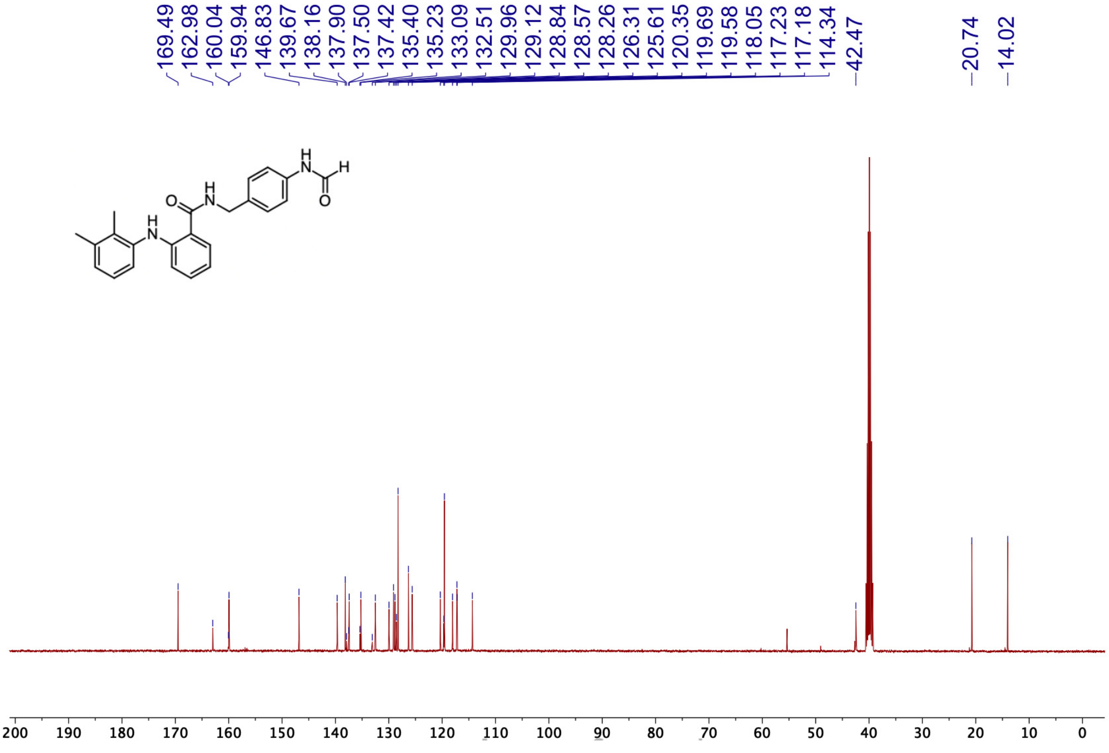

4h ^{1}H NMR

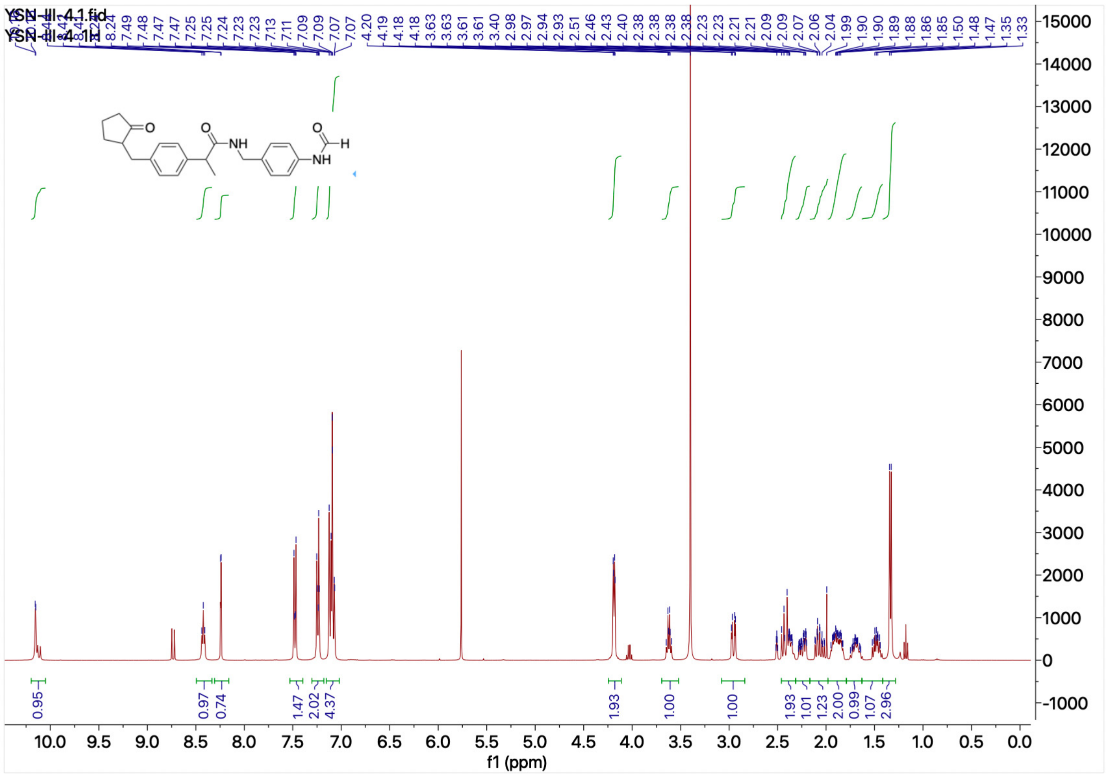

4h ^{13}C NMR

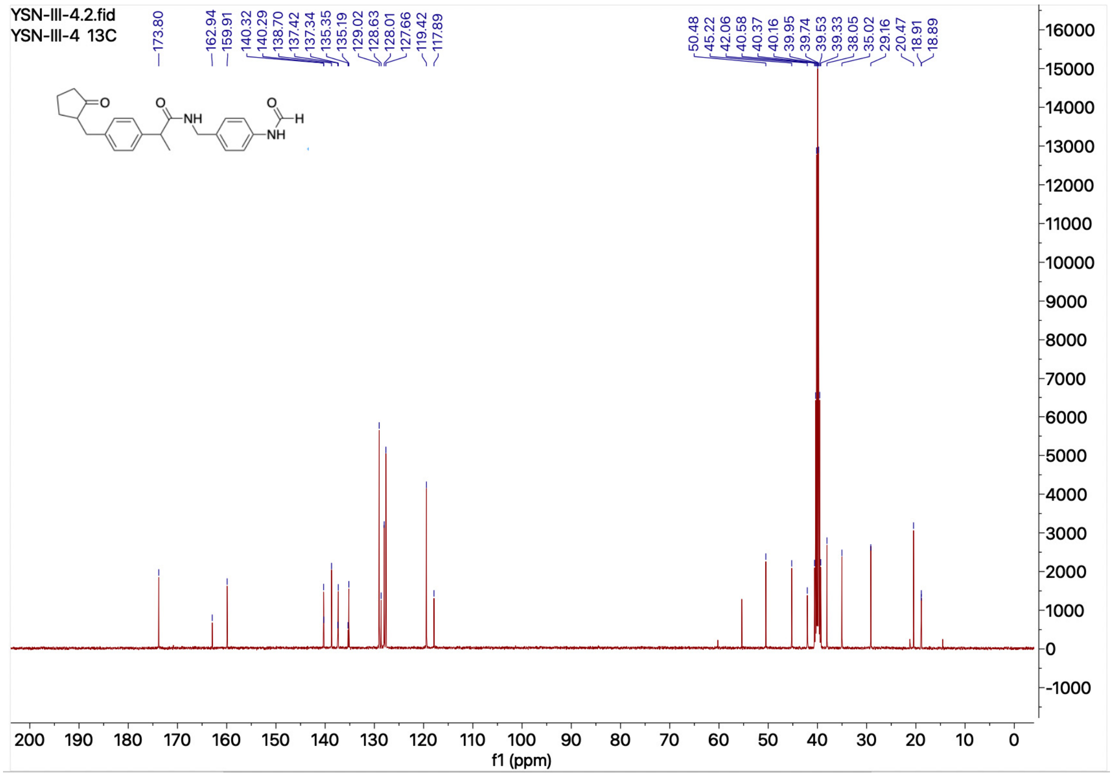

5a ^{1}H NMR

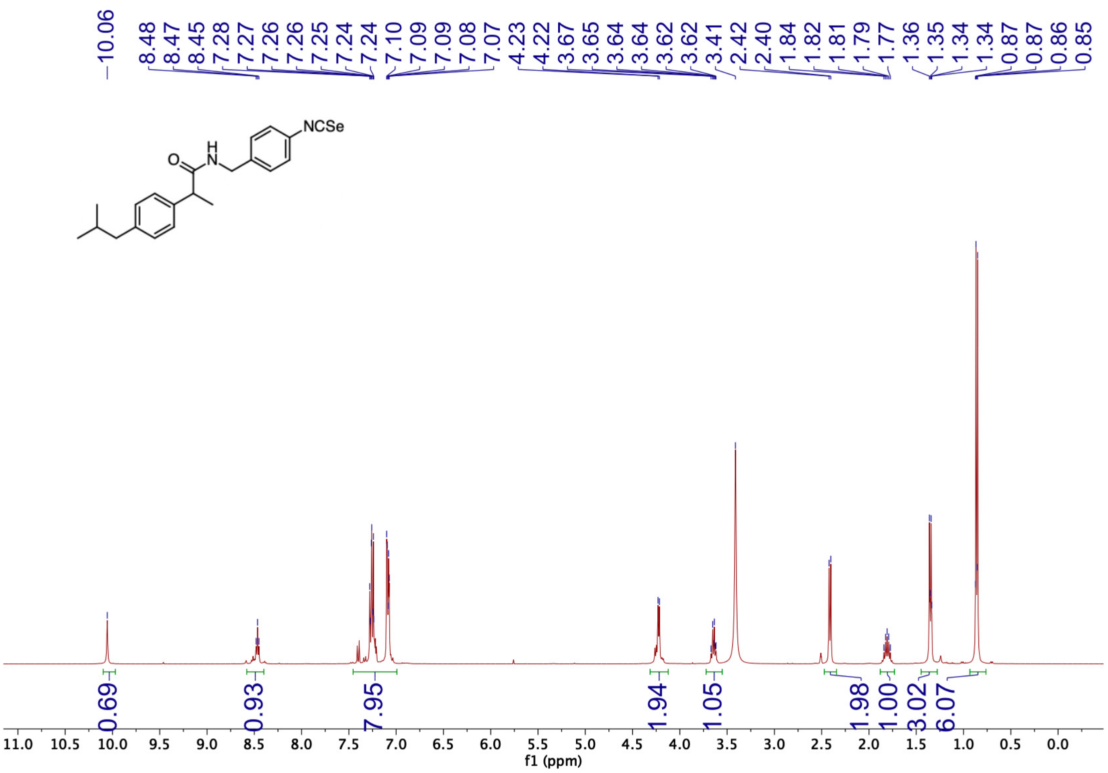

5a ^{13}C NMR

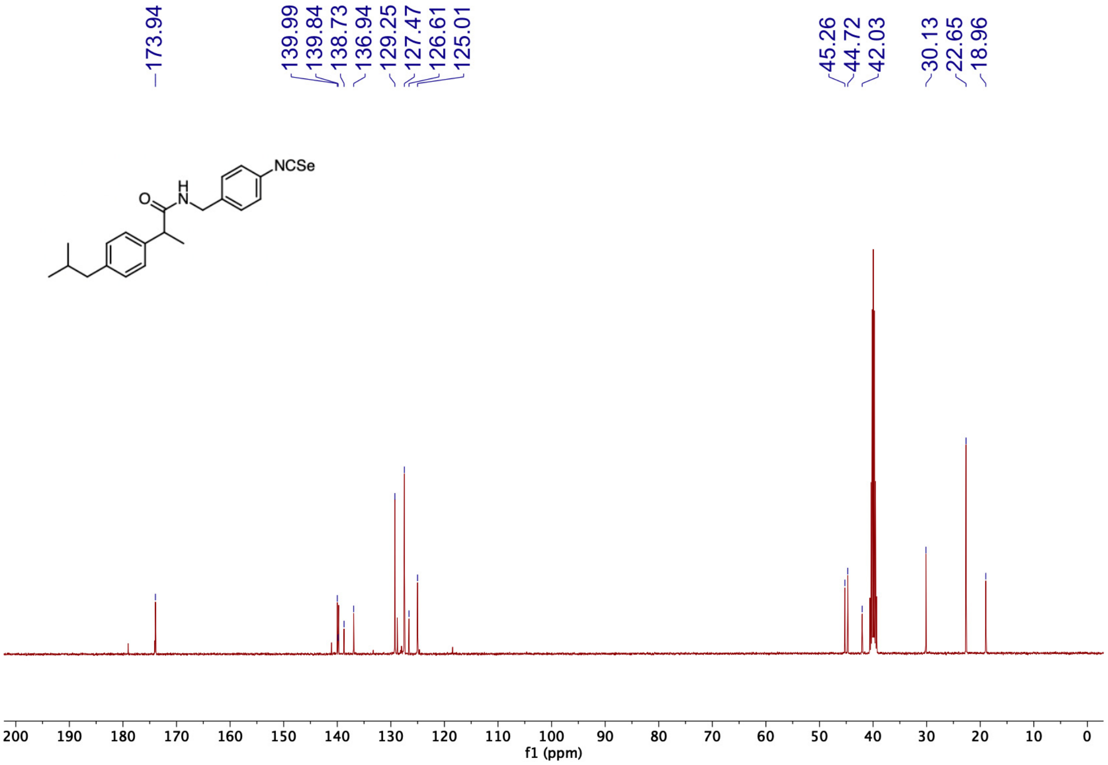

5b ^{1}H NMR

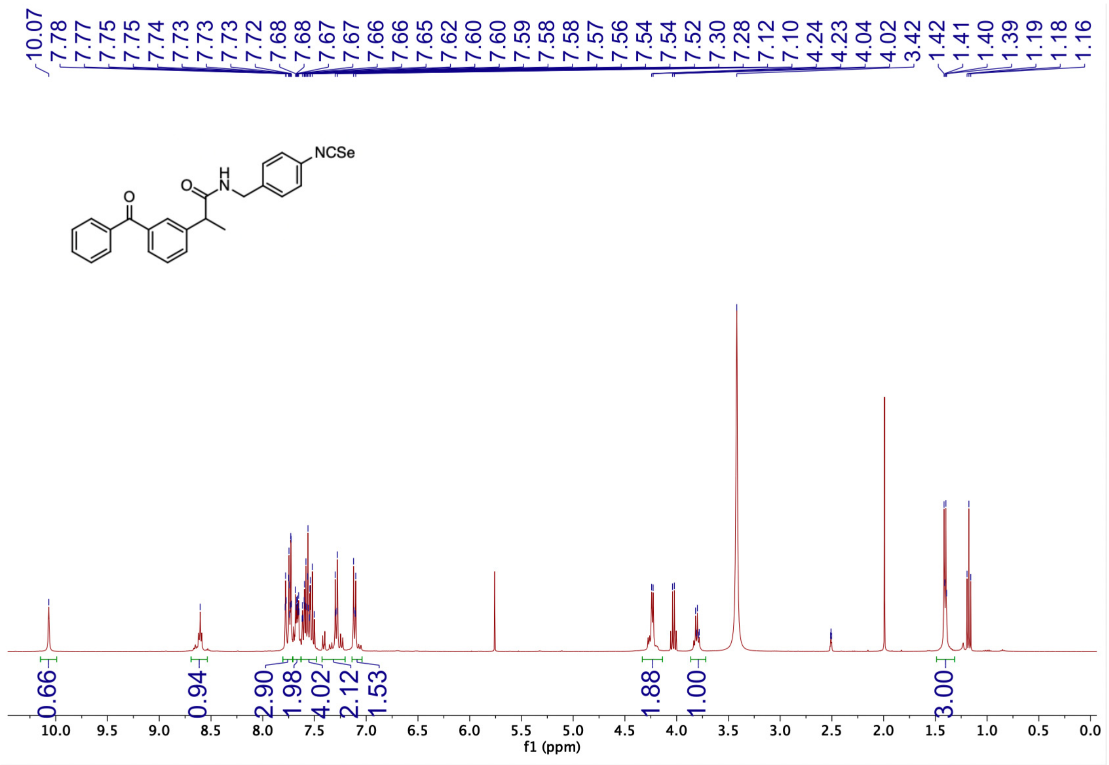

5b ^{13}C NMR

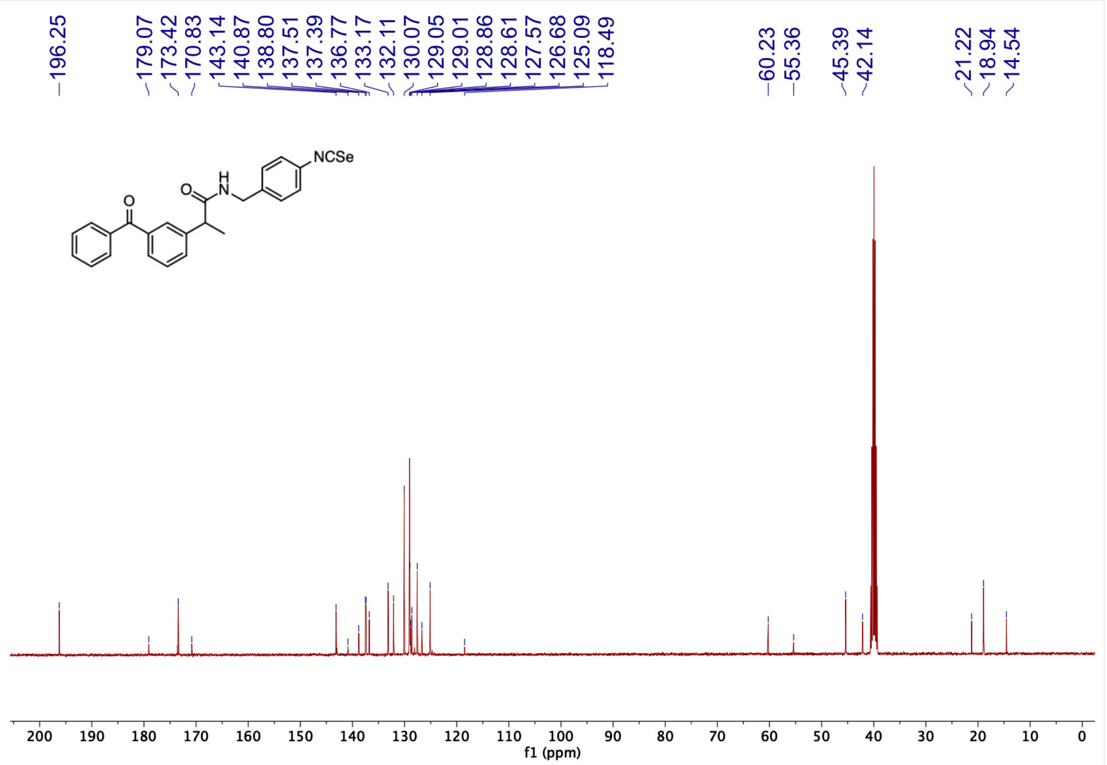

5c ^{1}H NMR

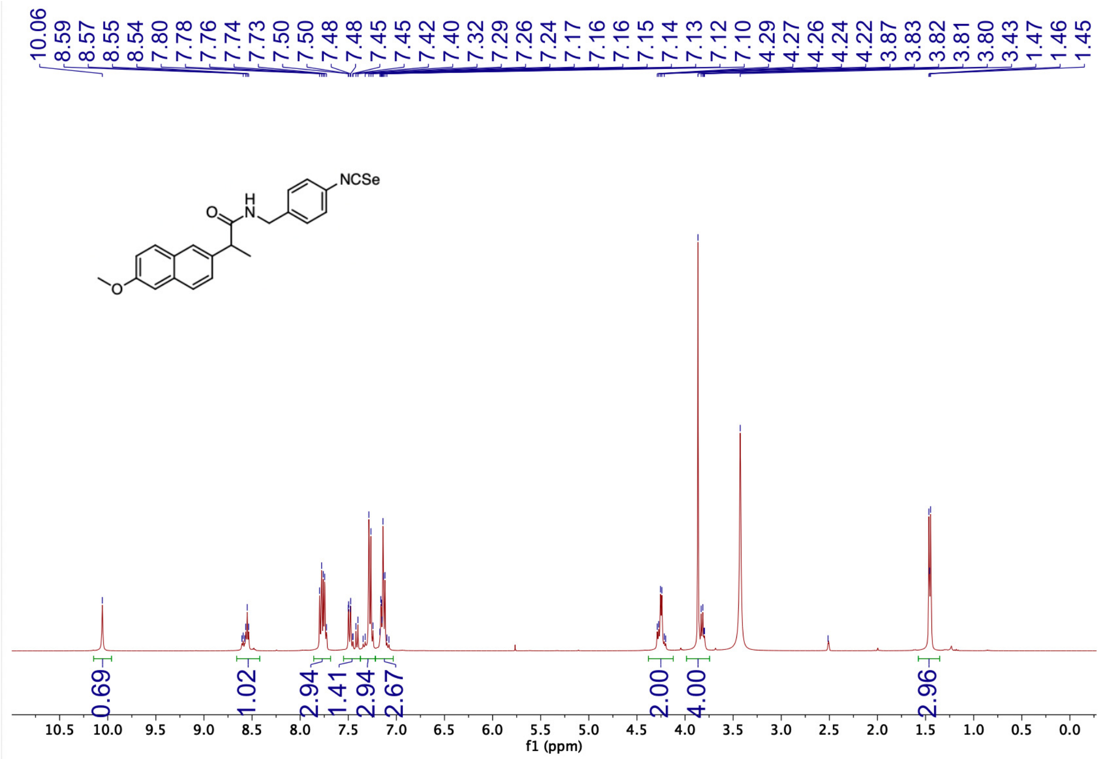

5c ^{13}C NMR

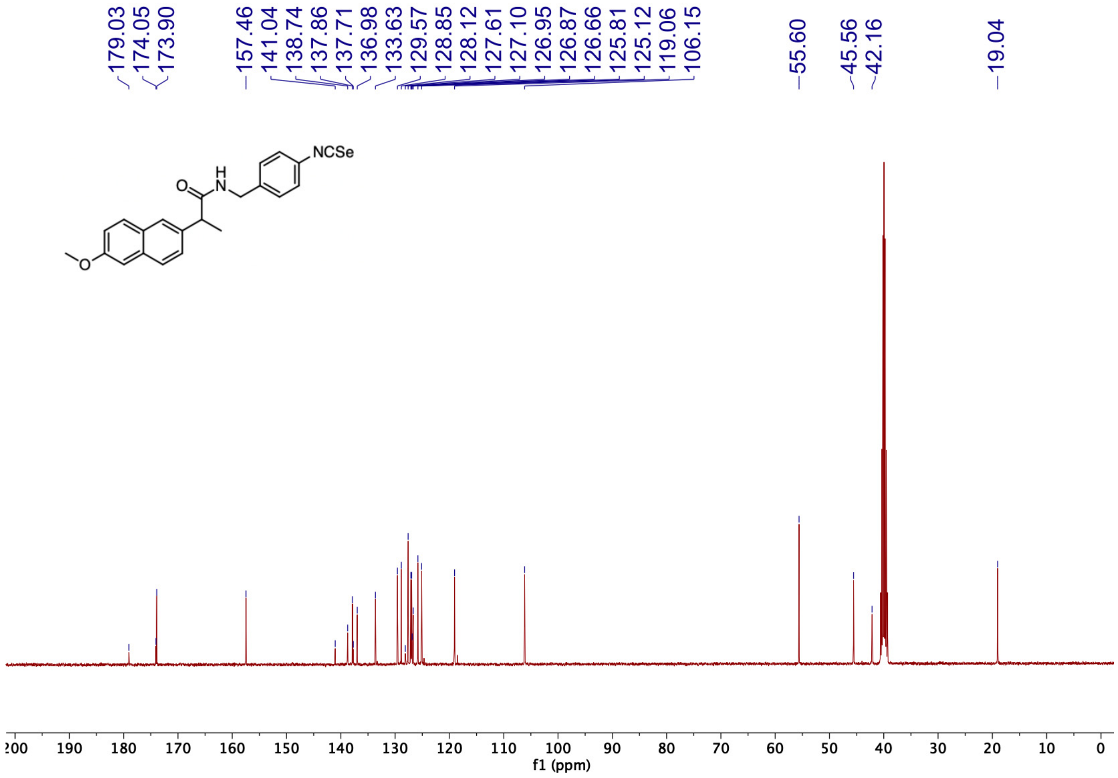

5d ^{1}H NMR

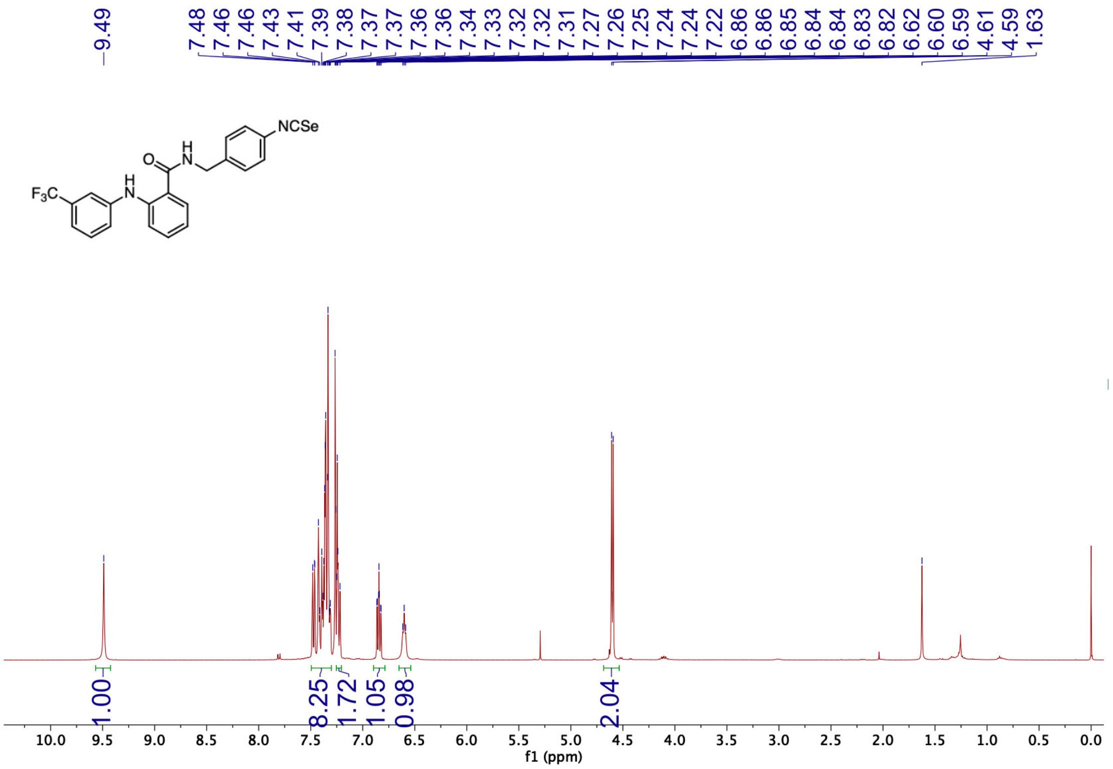

5d ^{13}C NMR

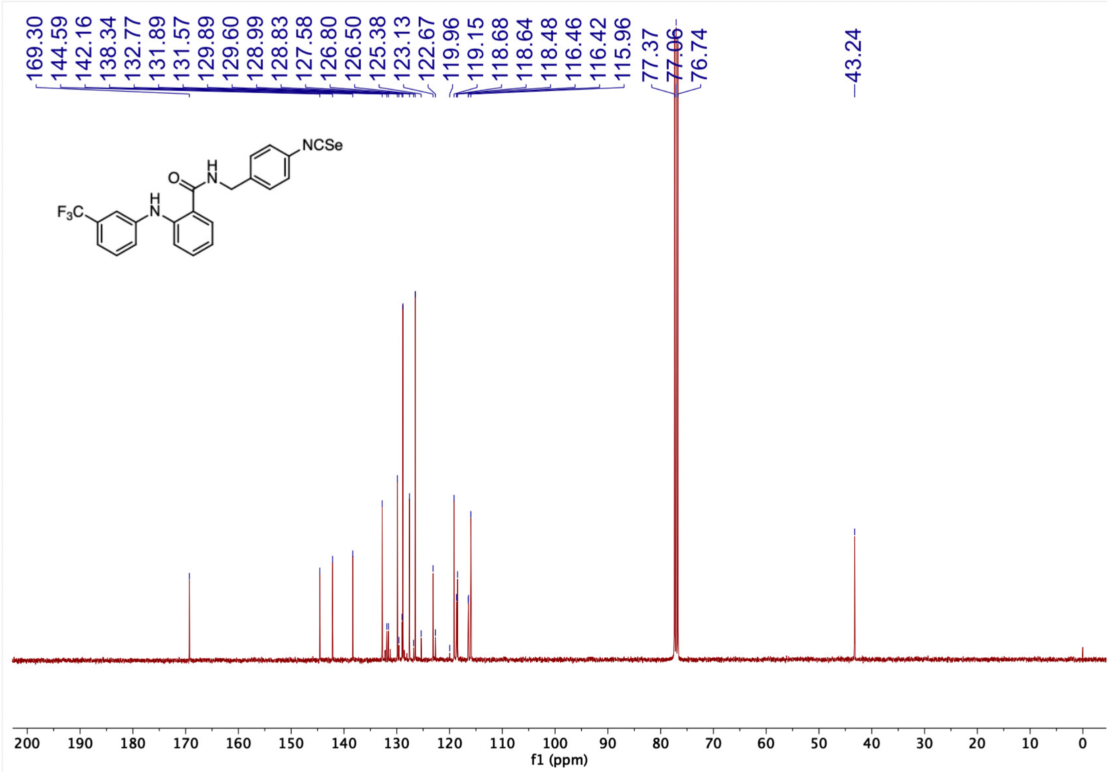

5e ¹H NMR

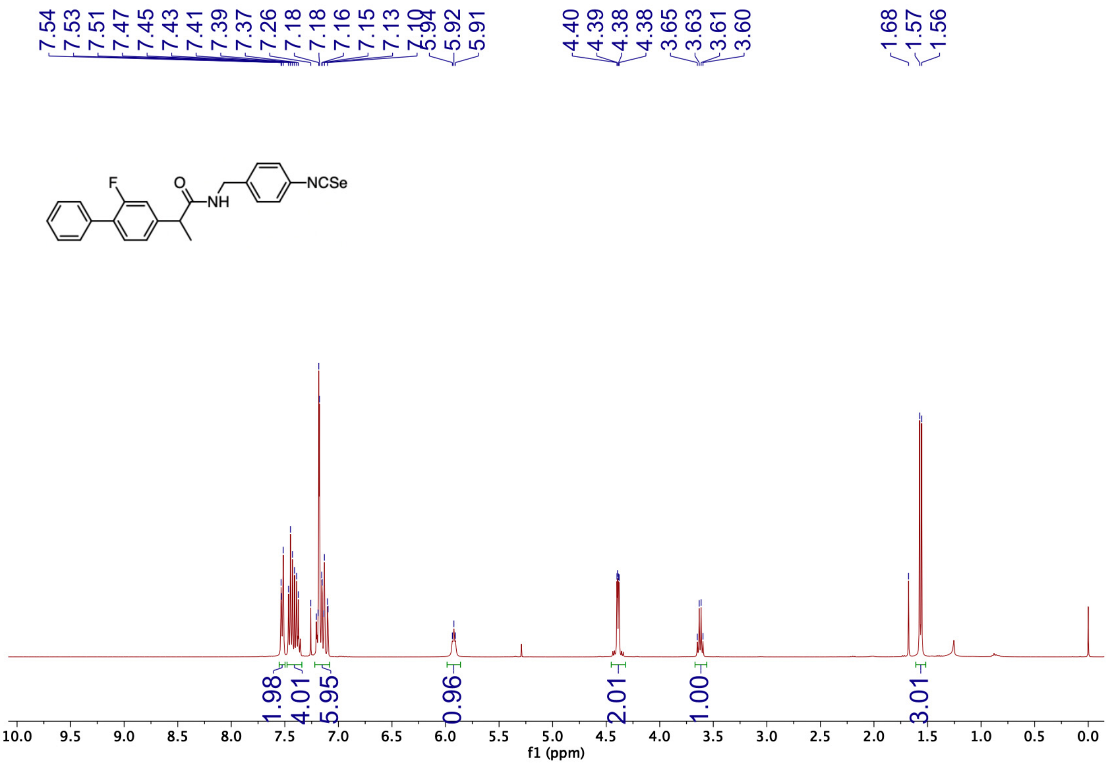

5e ¹³C NMR

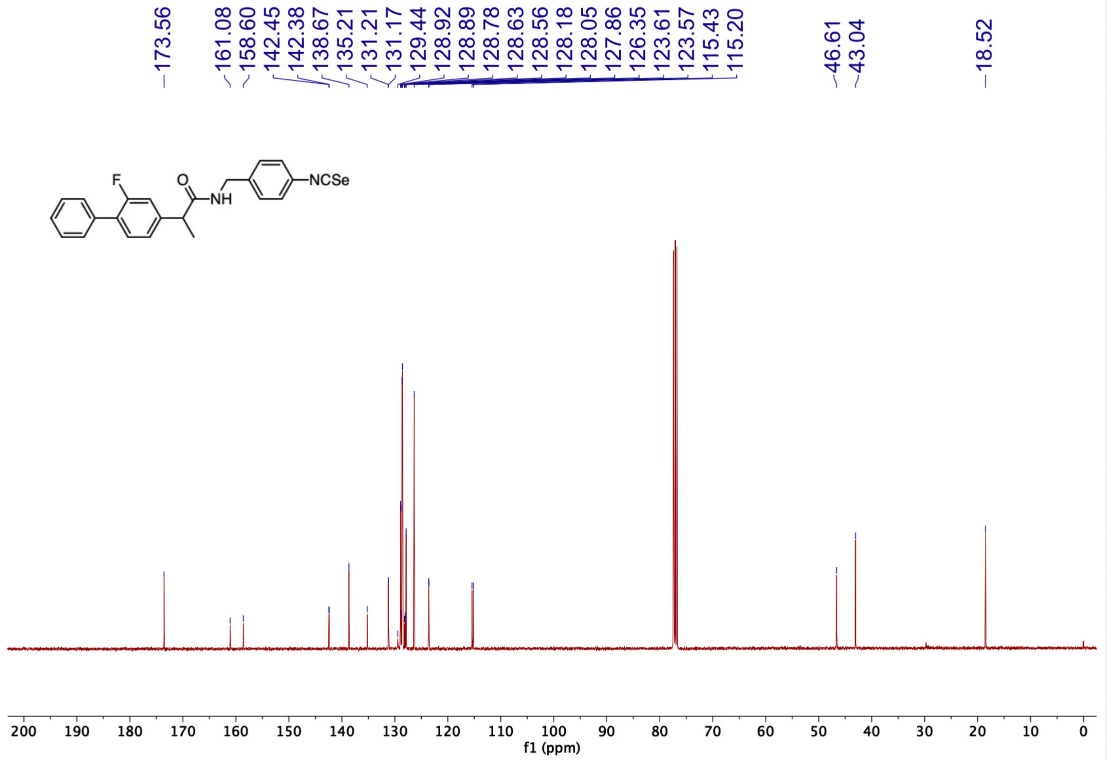

5f ^{1}H NMR

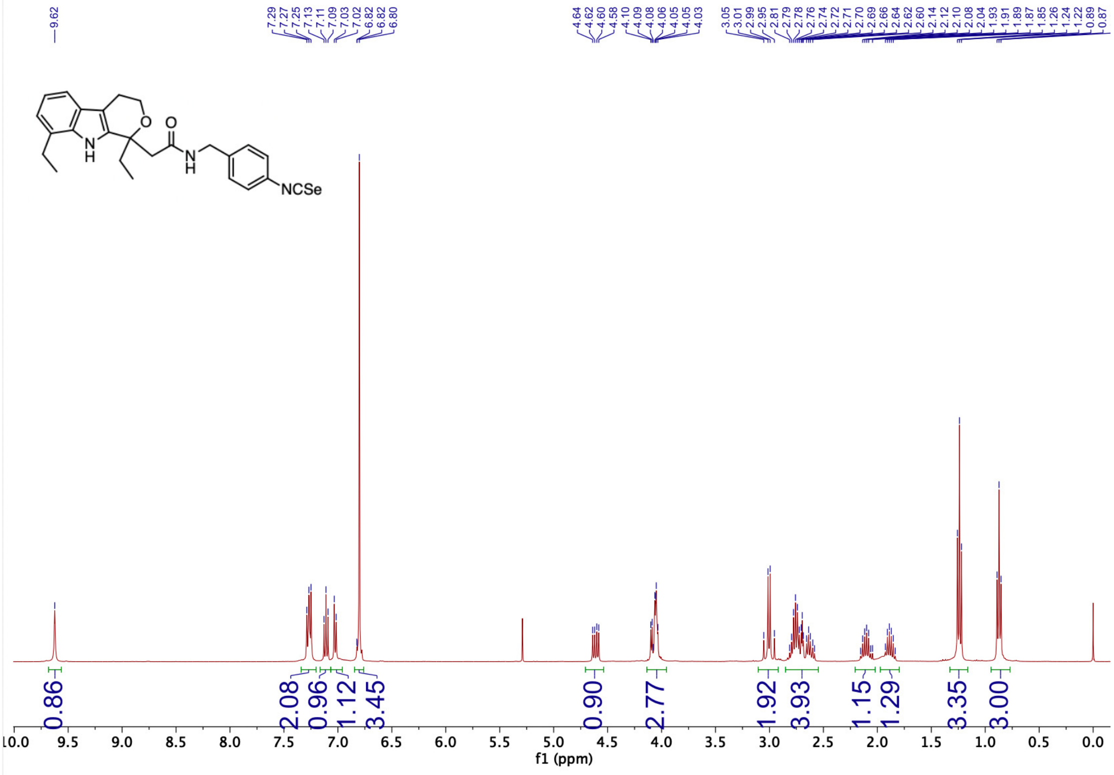

5f ^{13}C NMR

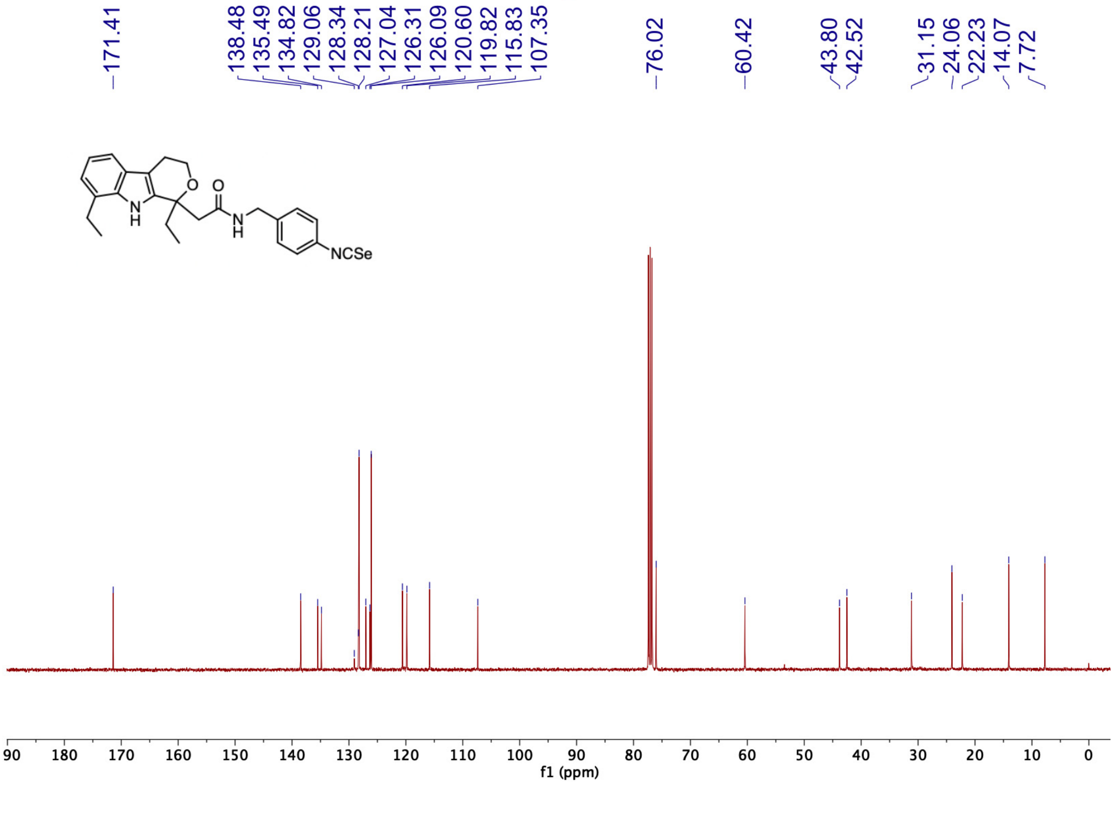

5g ^{1}H NMR

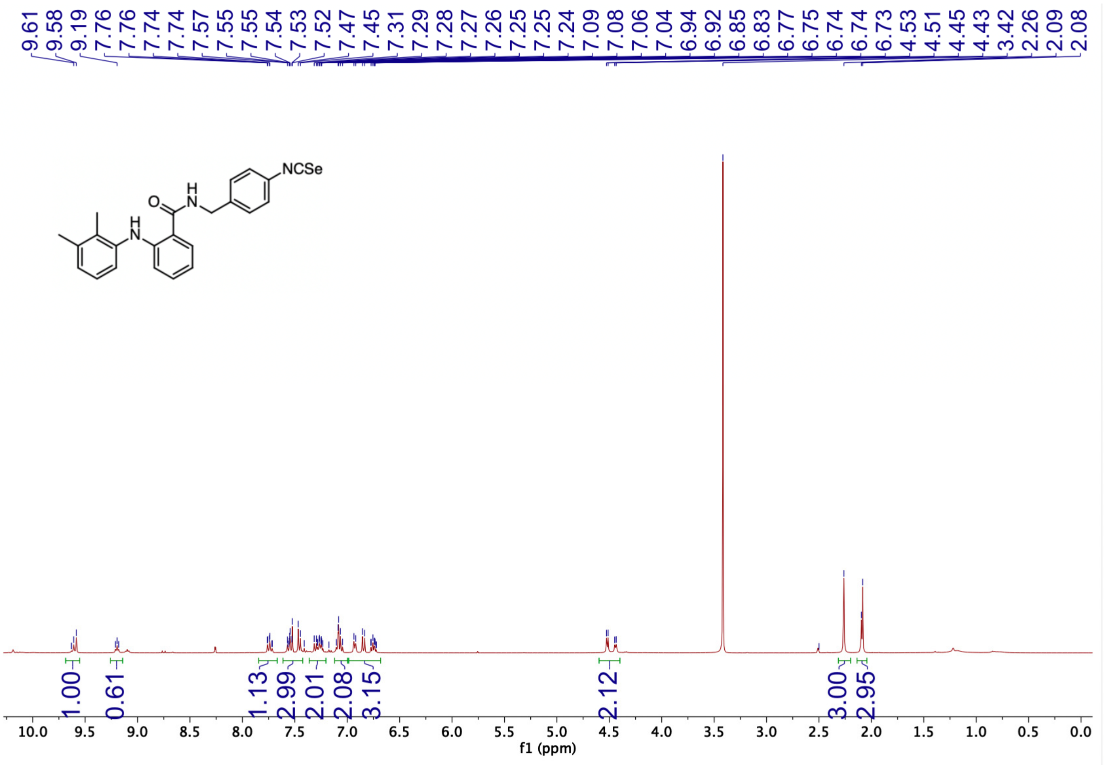

5g ^{13}C NMR

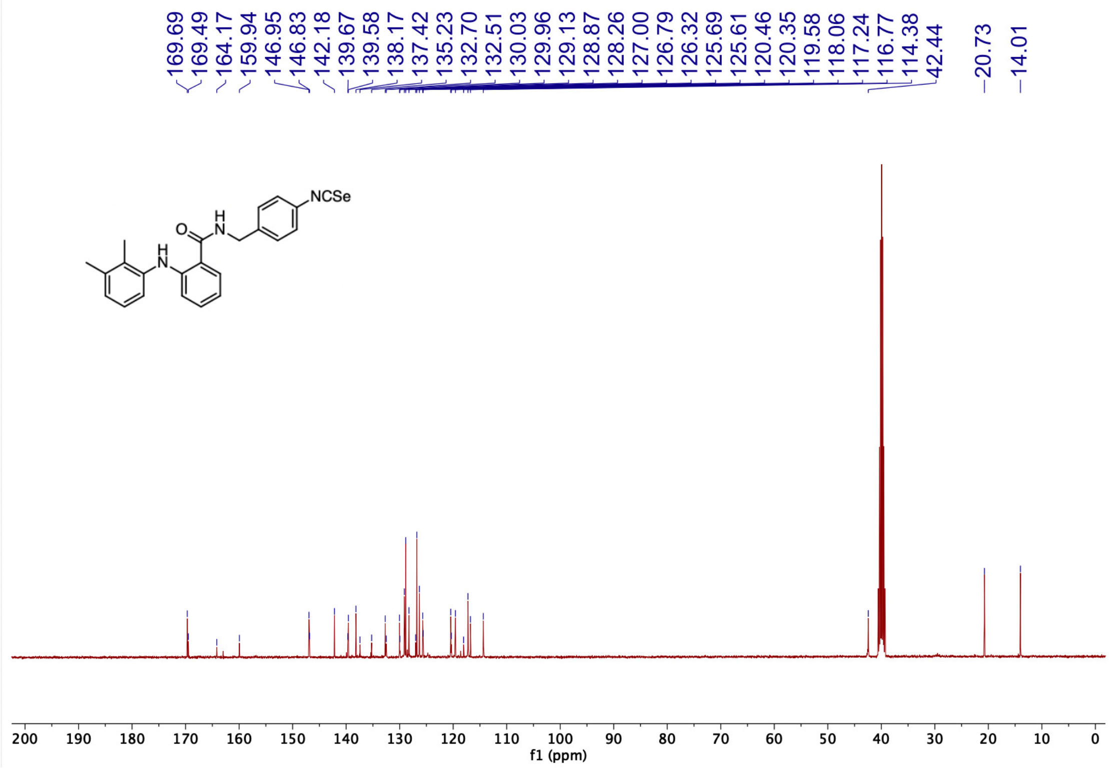

5h ^{1}H NMR

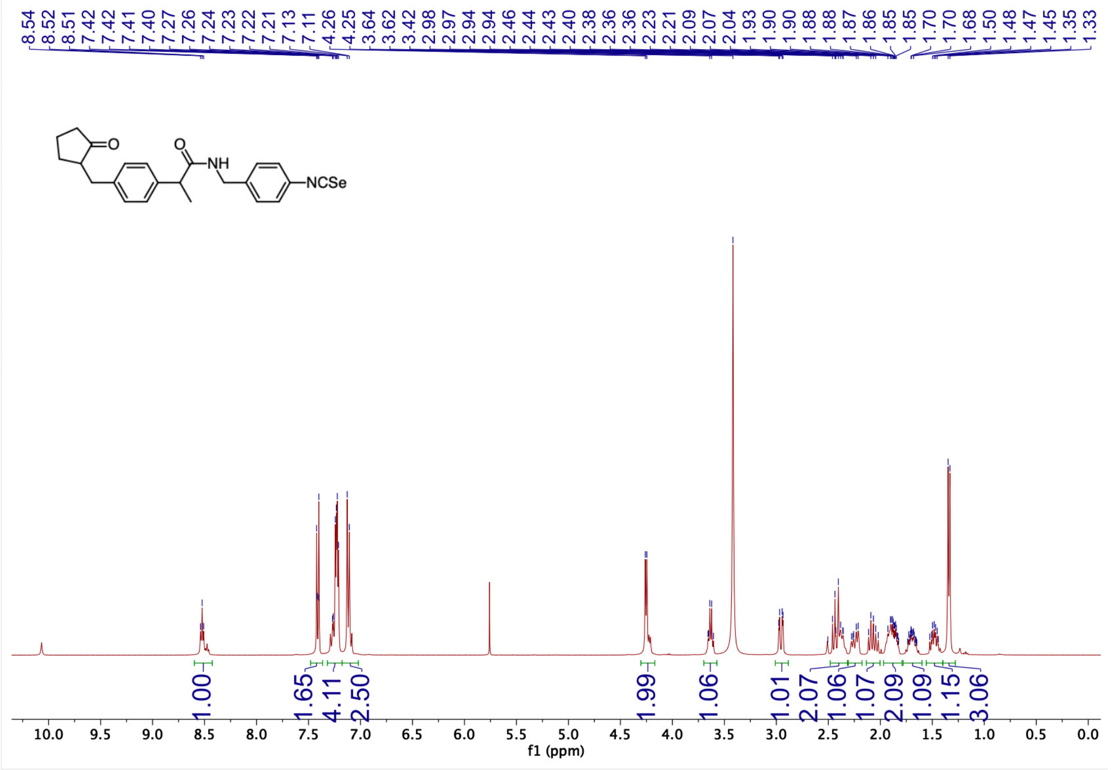

5h ^{13}C NMR

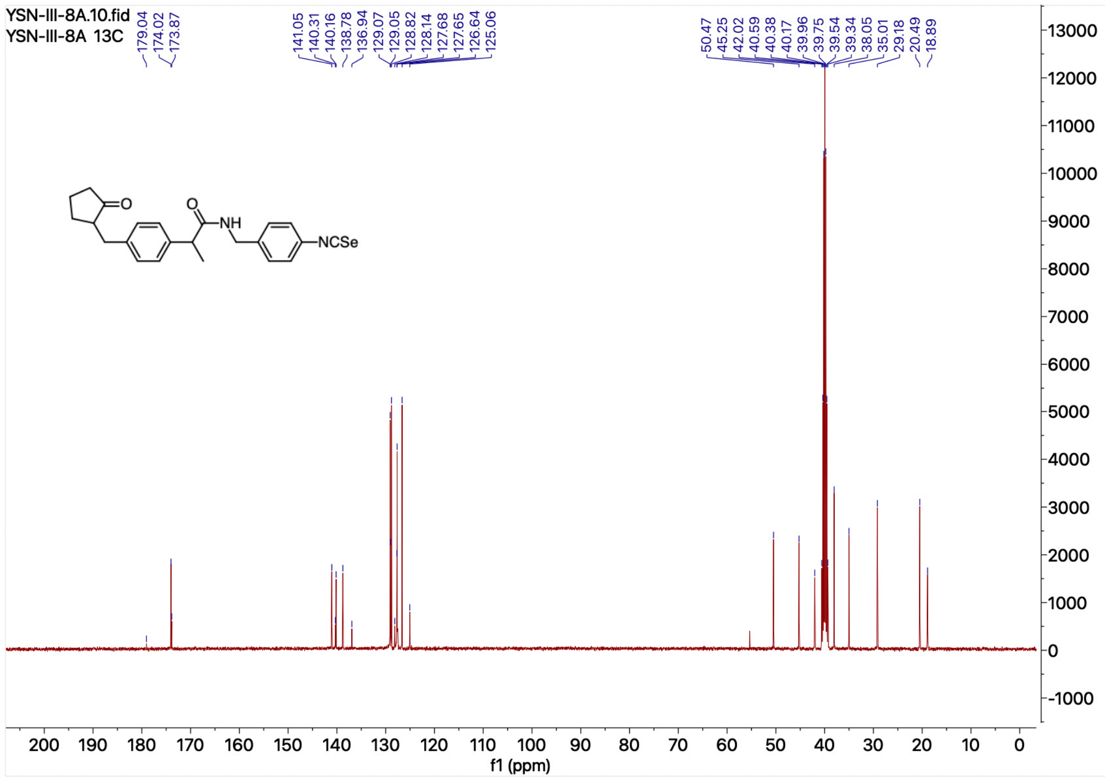

6a-^{1}H NMR

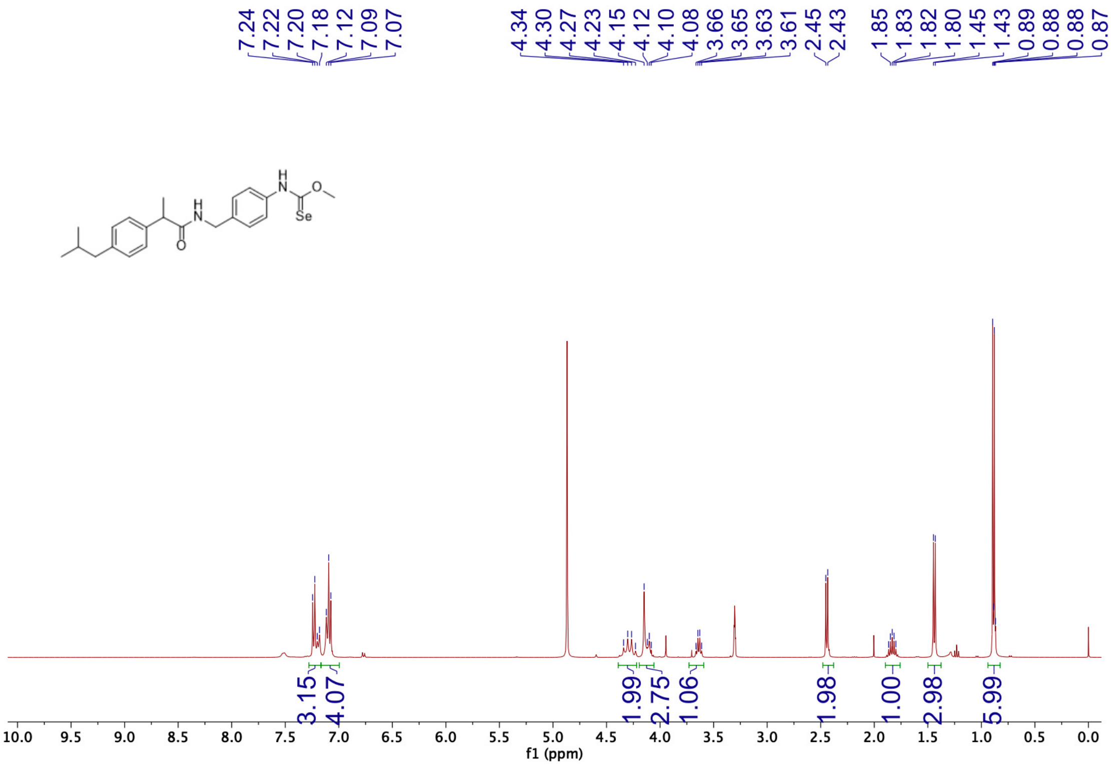

6a-^{13}C NMR

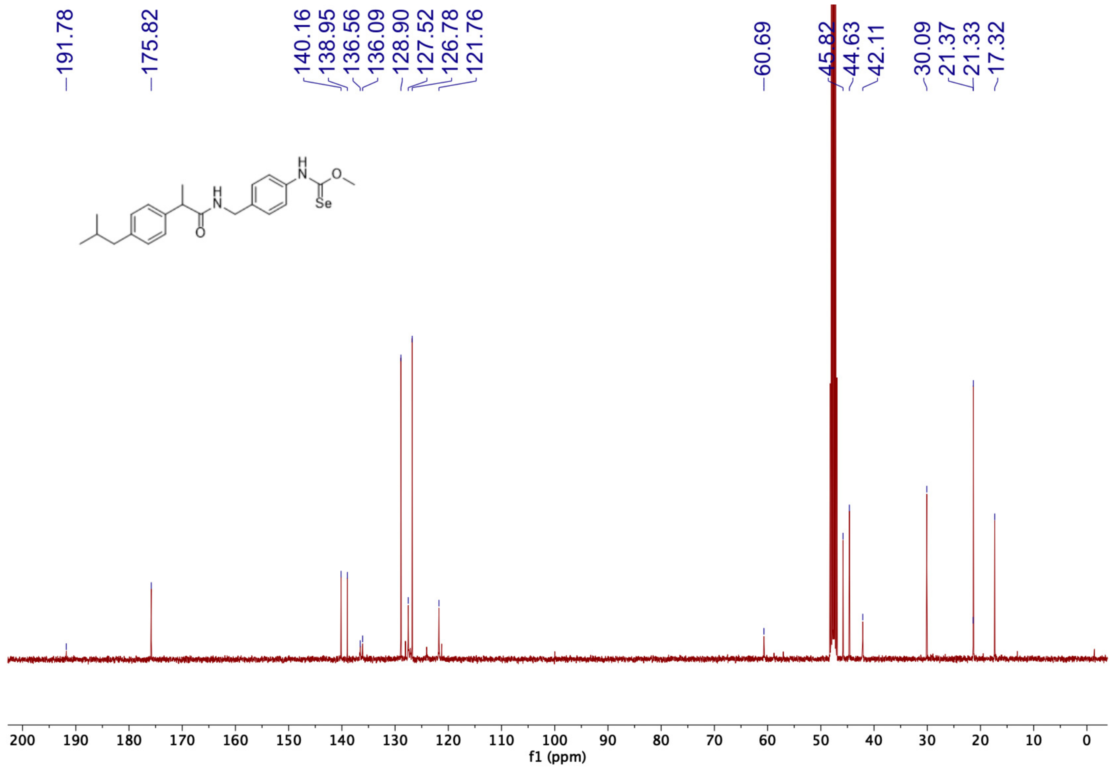

6b ^{1}H NMR

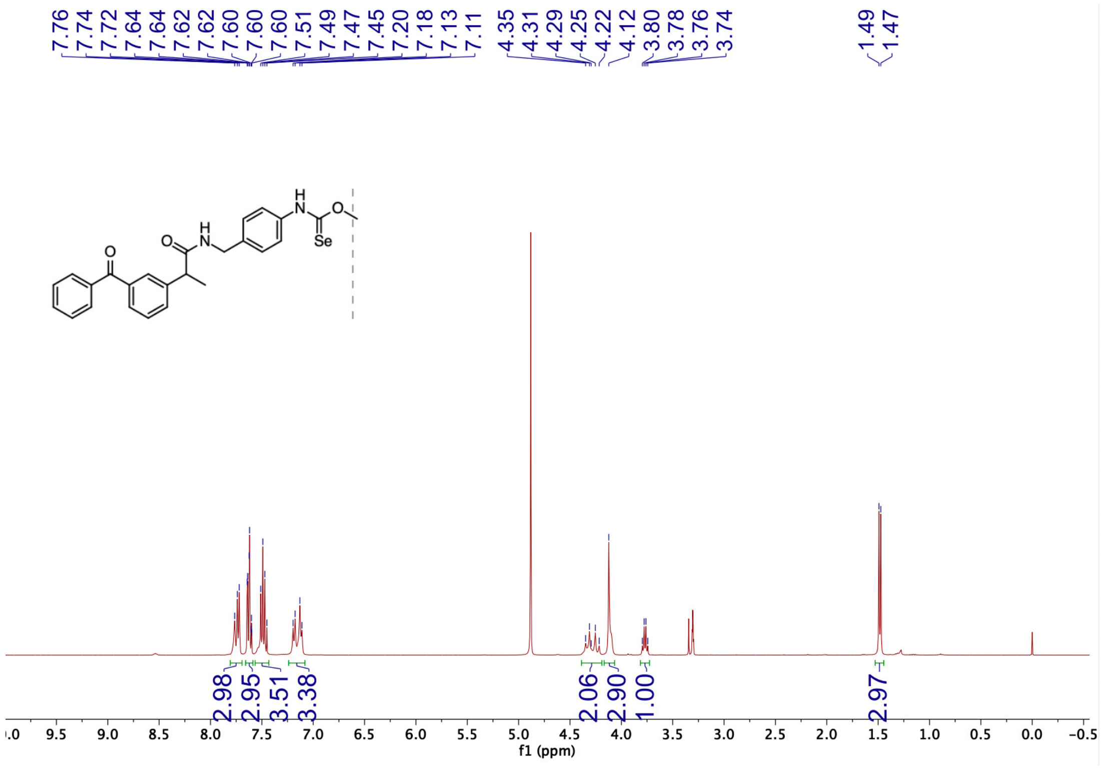

6b-^{13}C NMR

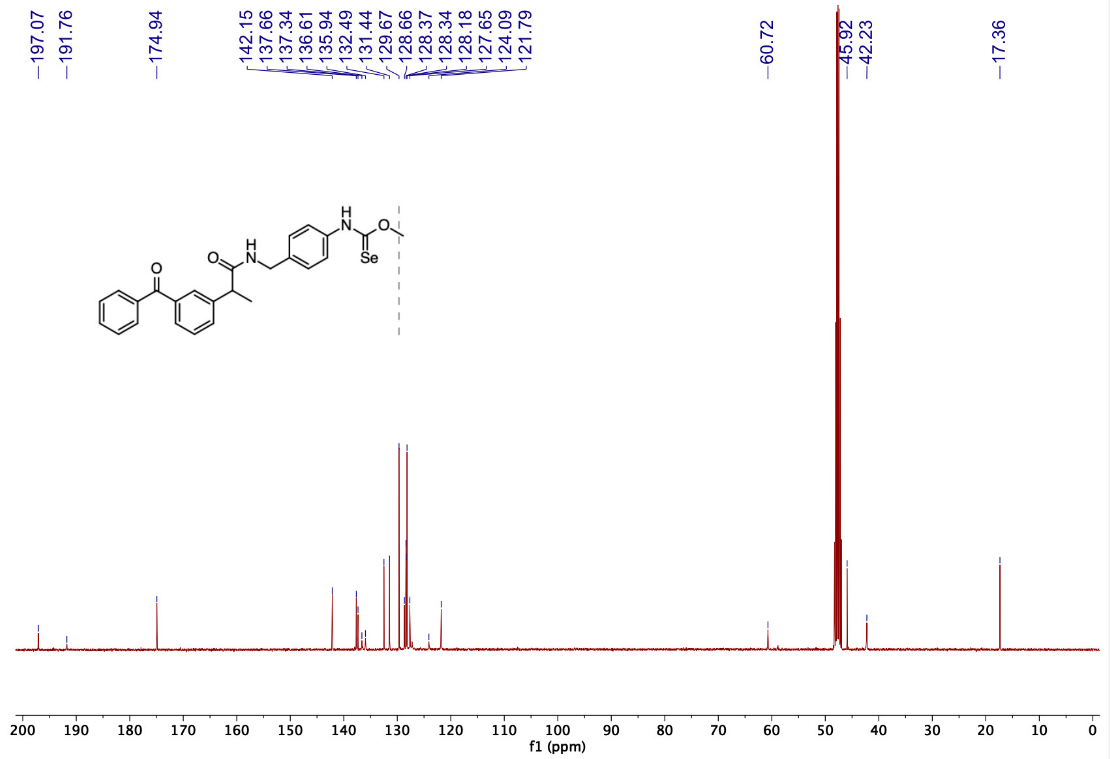

6c-^{1}H NMR

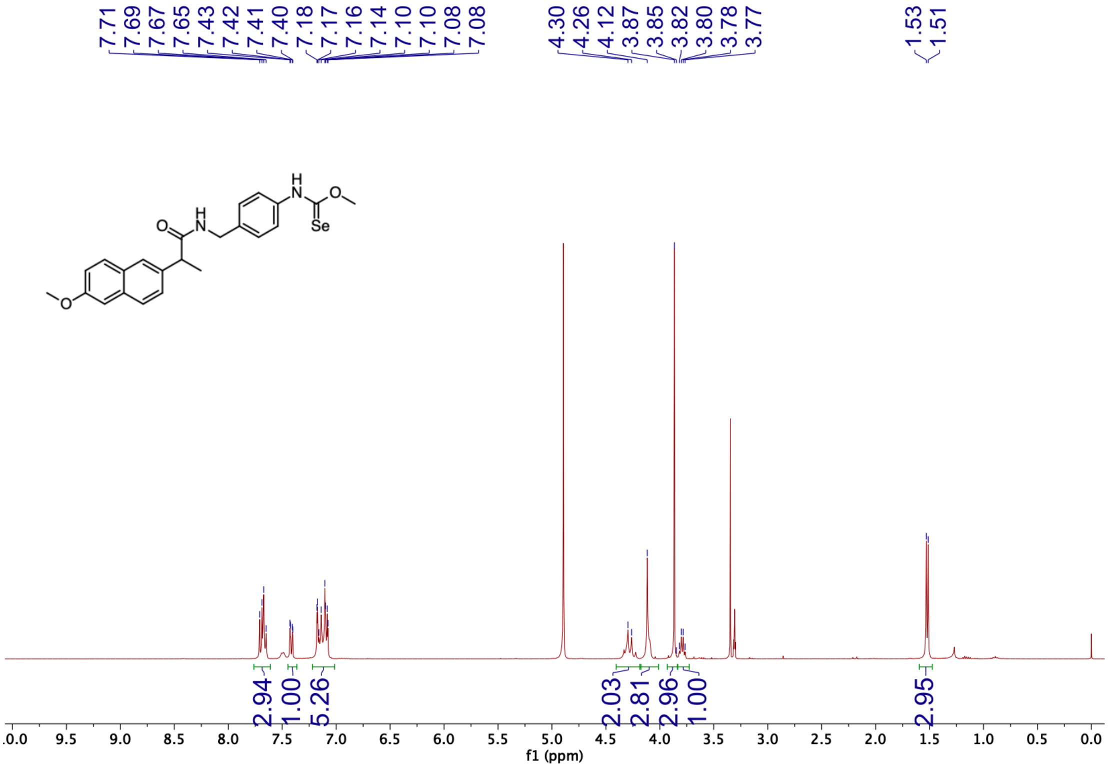

6c-^{13}C NMR

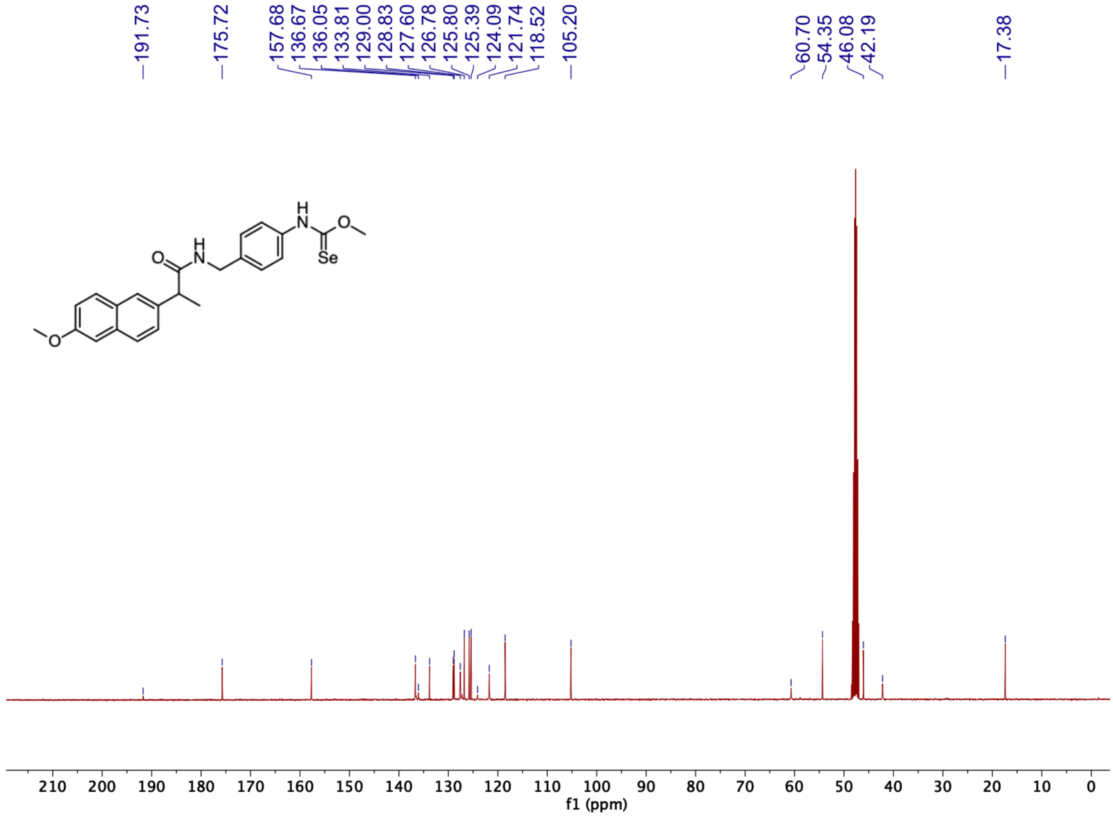

6d-^{1}H NMR

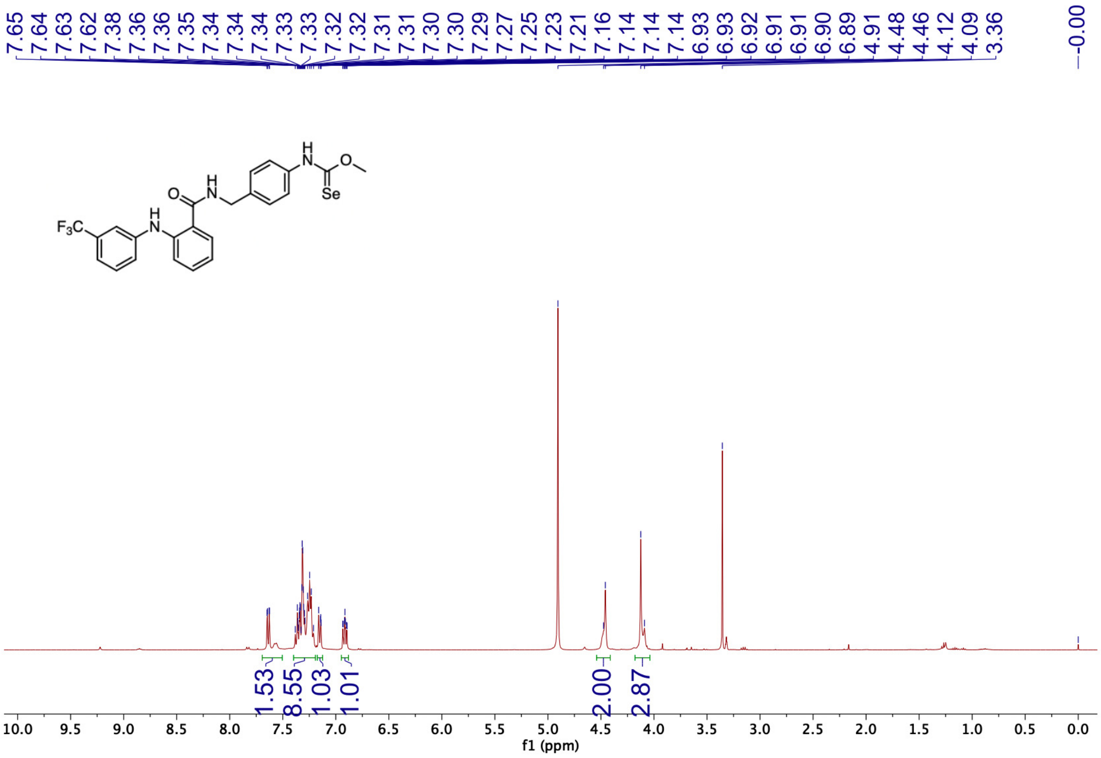

6d-^{13}C NMR

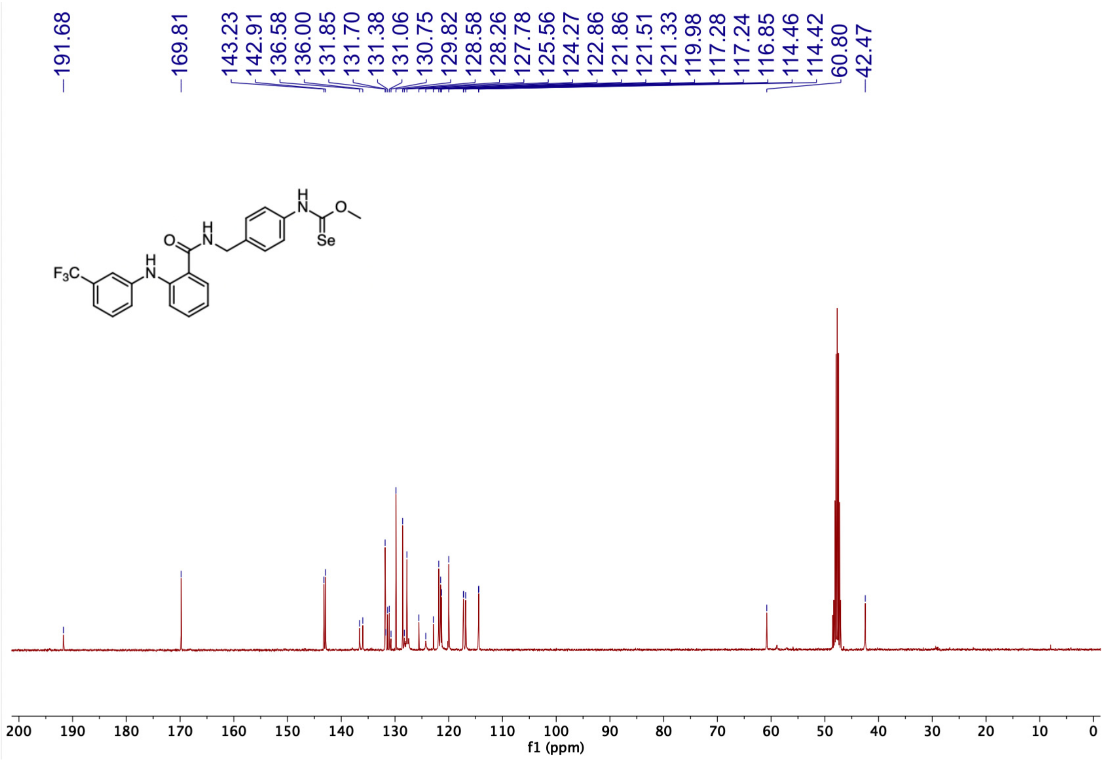

6e-^{1}H NMR

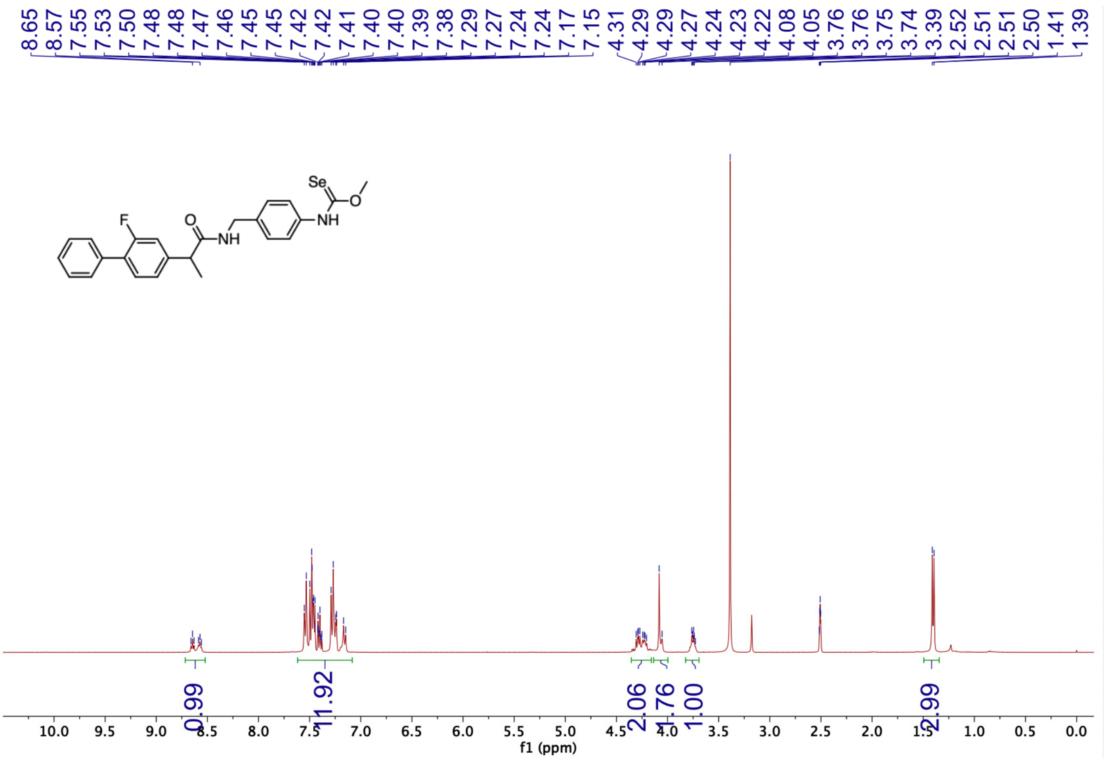

6e-^{13}C NMR

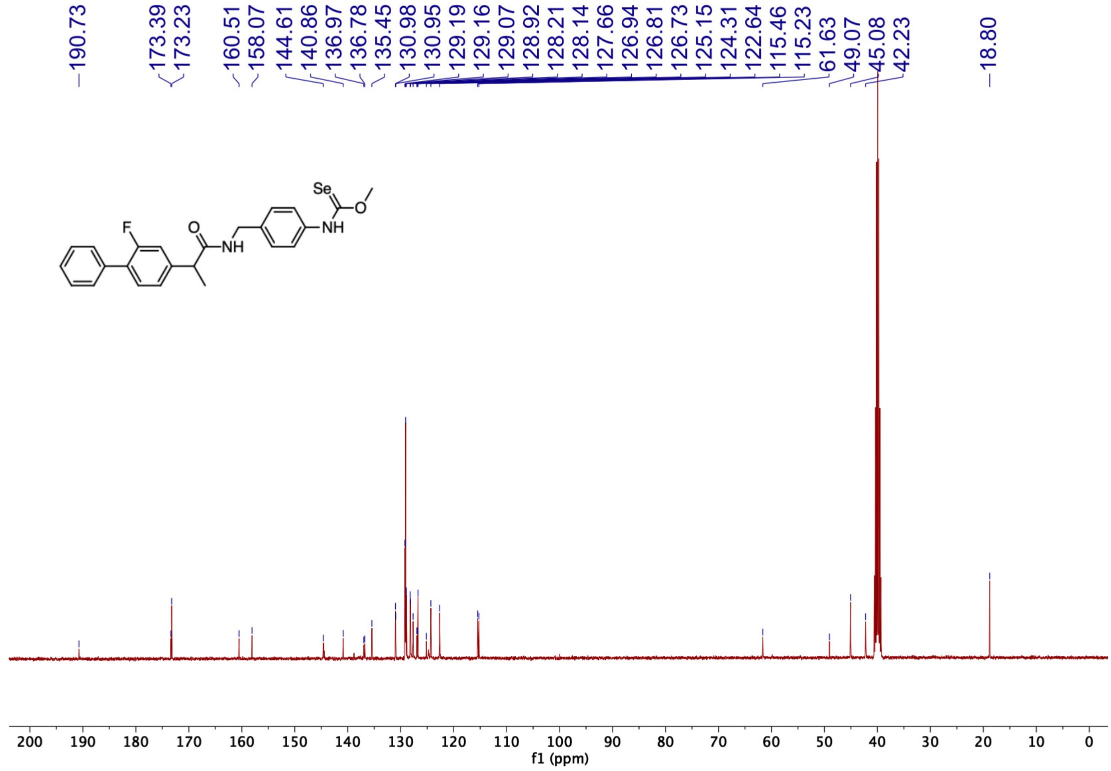

6f-^{1}H NMR

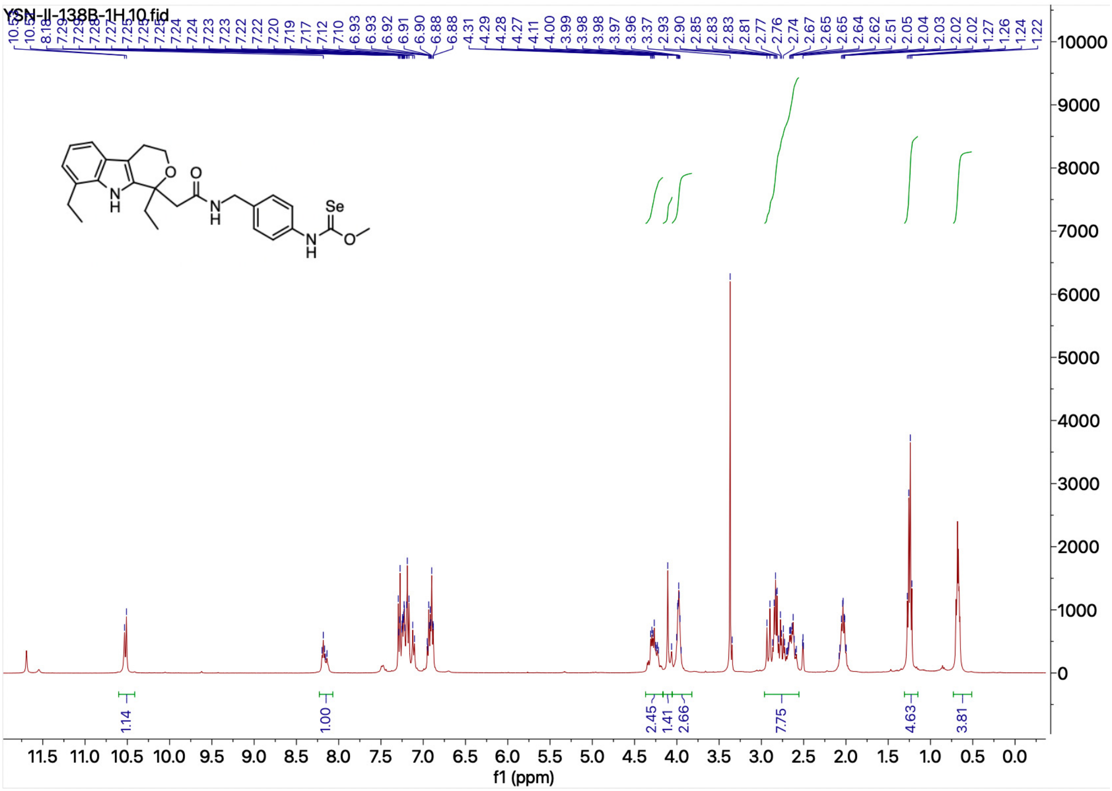

6f-^{13}C NMR

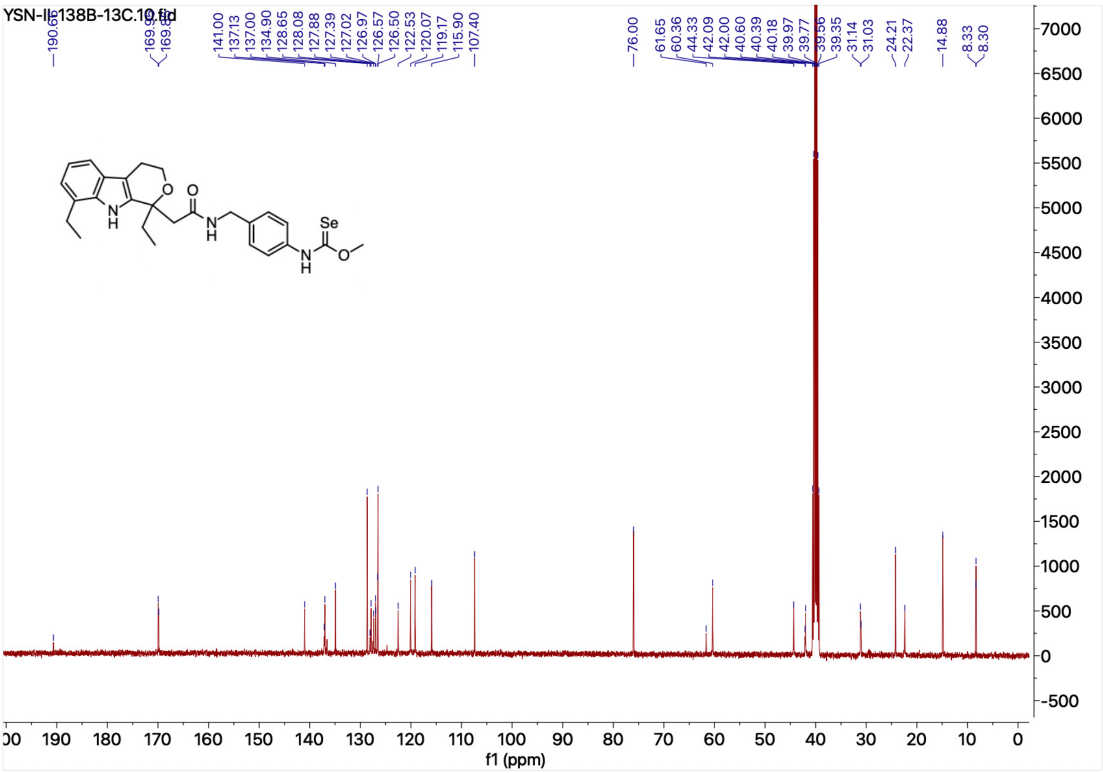

6g ¹H NMR

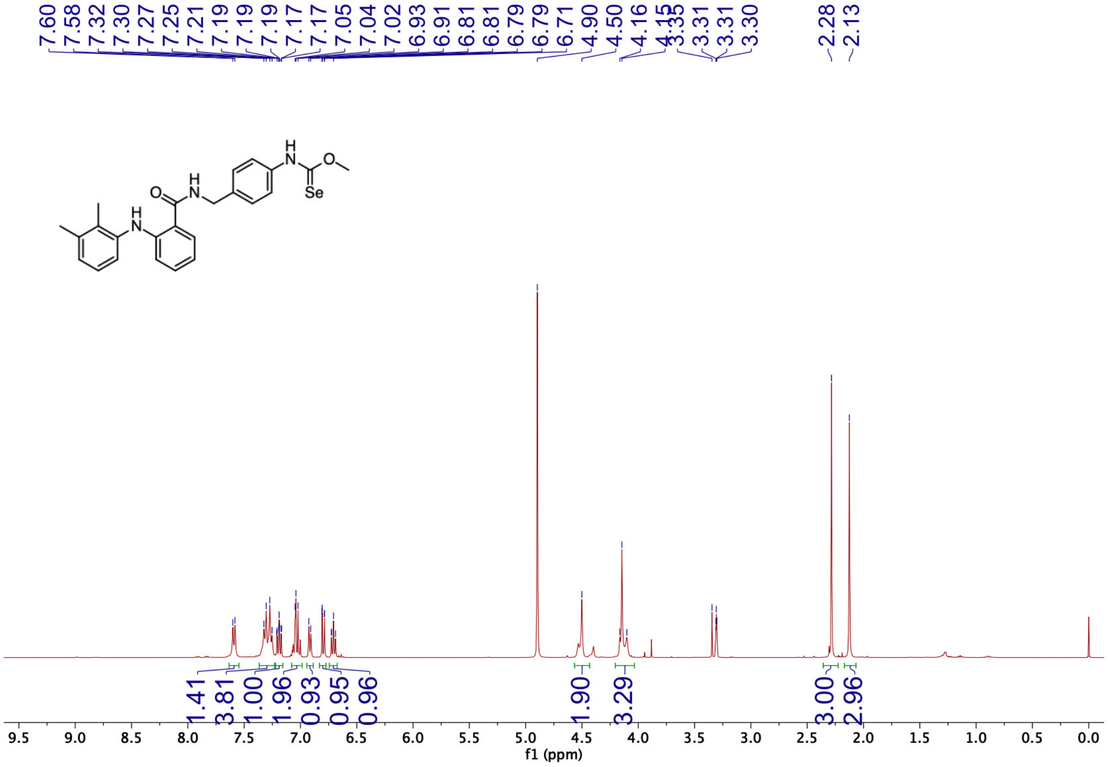

6g ¹³C NMR

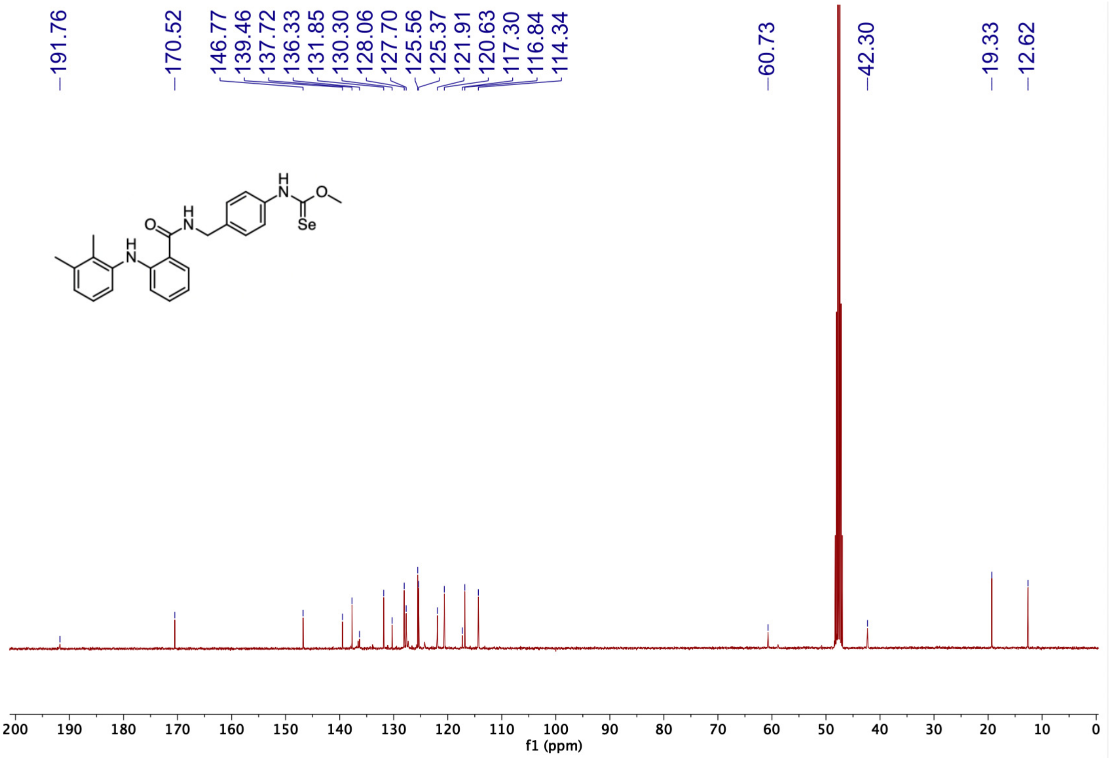

6h ^{1}H NMR

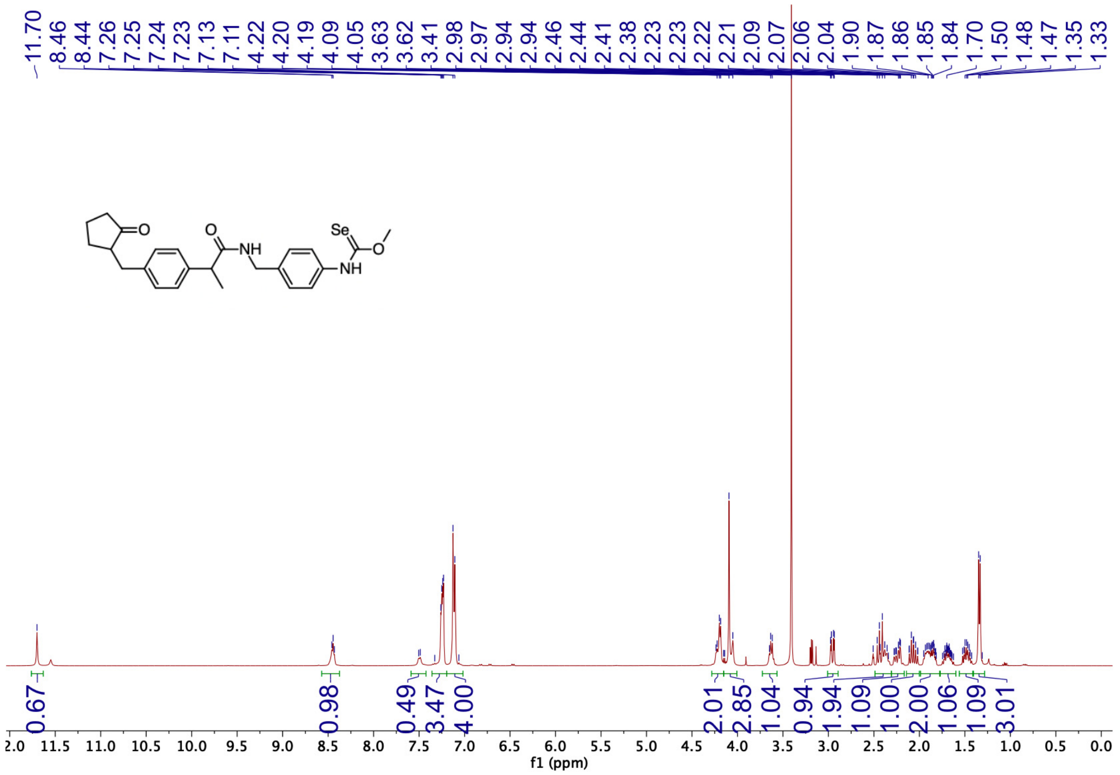

6h ^{13}C NMR

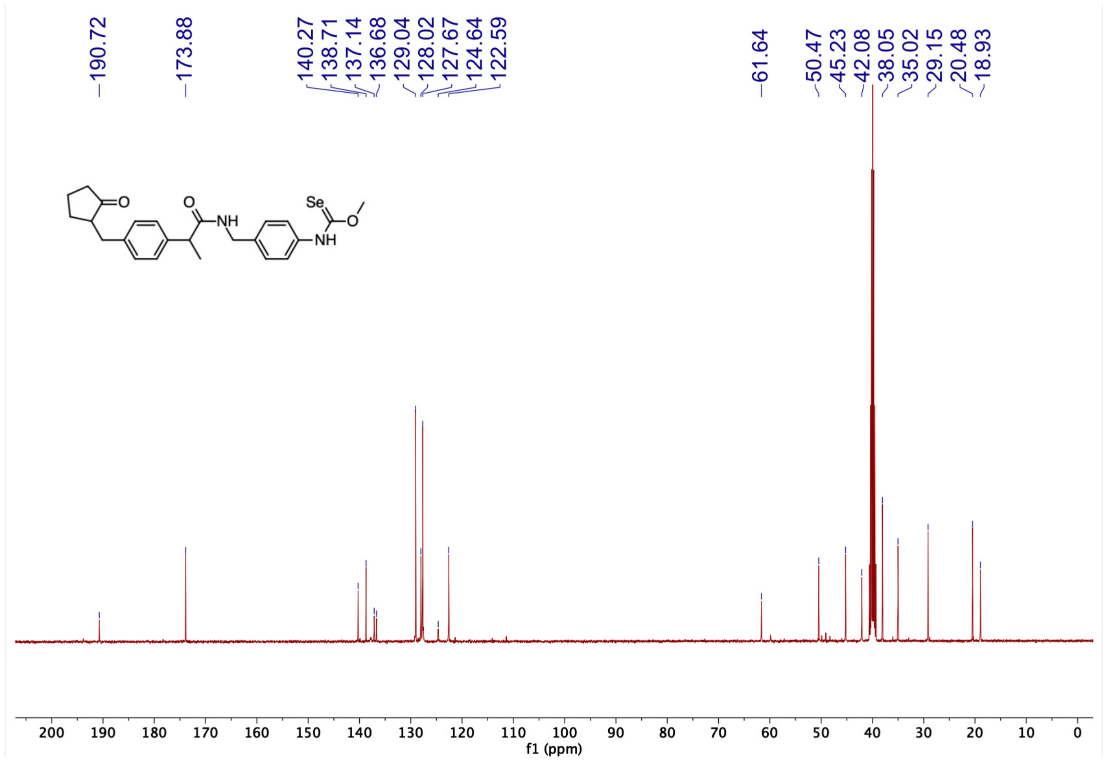

5a MS

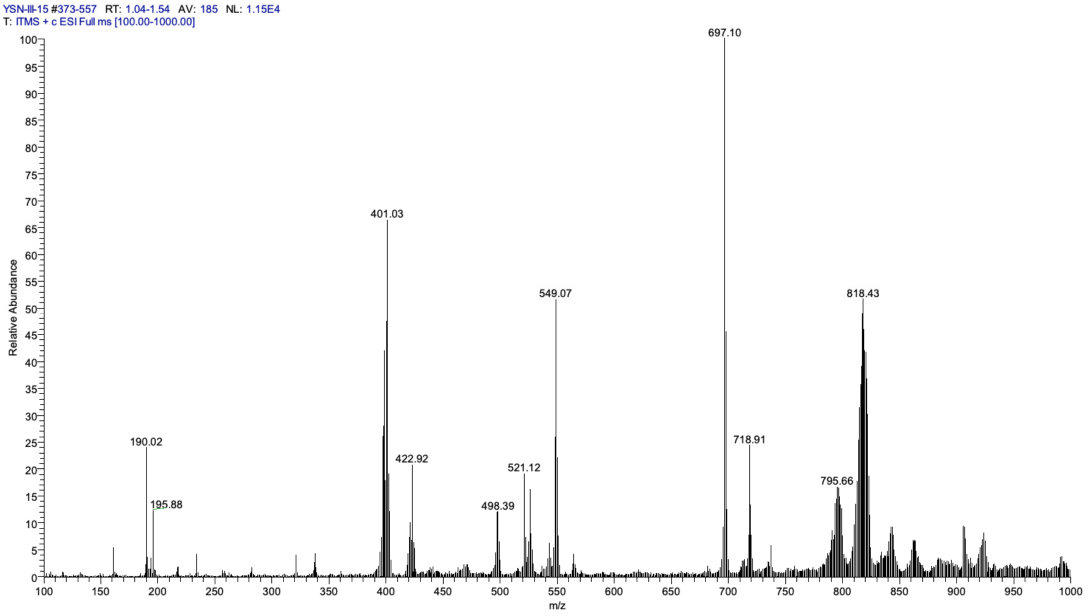
YSN-III-15 #373-557 RT: 1.04-1.54 AV: 185 NL: 1.15E4
T: ITMS + c ESI Full ms [100.00-1000.00]
697.10
401.03
549.07
818.43
190.02
195.88
422.92
521.12
498.39
718.91
795.66
Relative Abundance
m/z

5b MS

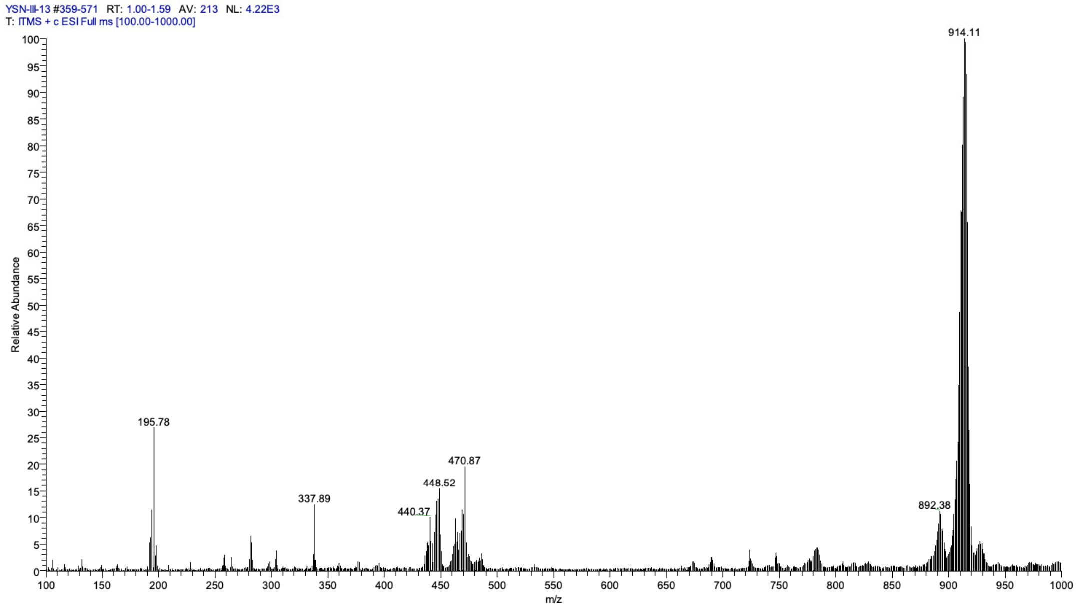
YSN-III-13 #359-571 RT: 1.00-1.59 AV: 213 NL: 4.22E3
T: ITMS + c ESI Full ms [100.00-1000.00]
914.11
195.78
470.87
448.52
337.89
440.37
892.38
Relative Abundance
m/z

5c MS

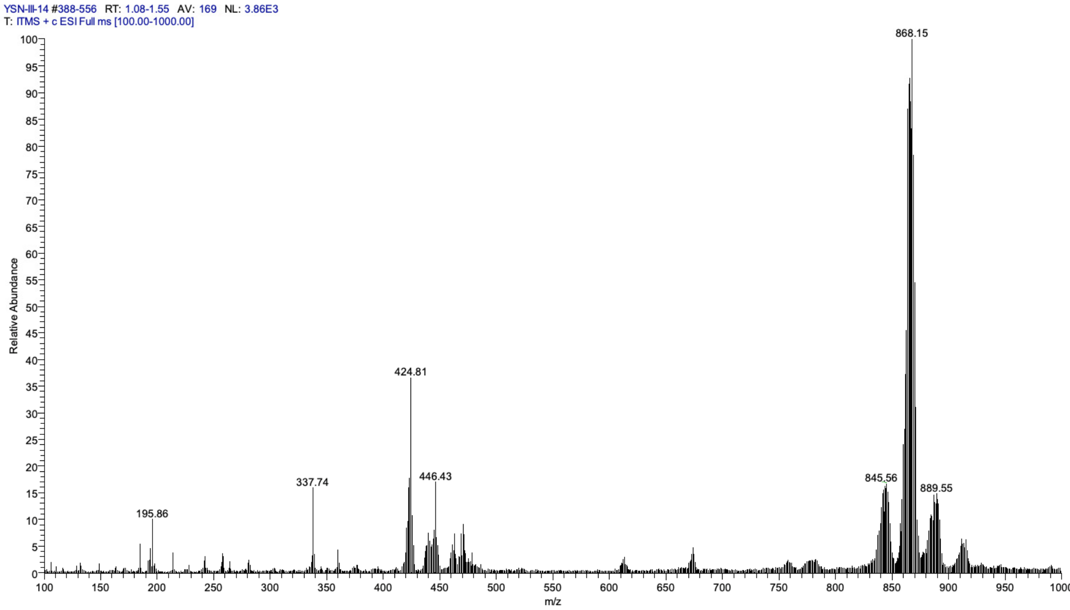
YSN-III-14 #388-556 RT: 1.08-1.55 AV: 169 NL: 3.86E3
T: ITMS + c ESI Full ms [100.00-1000.00]
868.15
424.81
337.74
446.43
845.56
889.55
195.86
Relative Abundance
m/z

5d MS

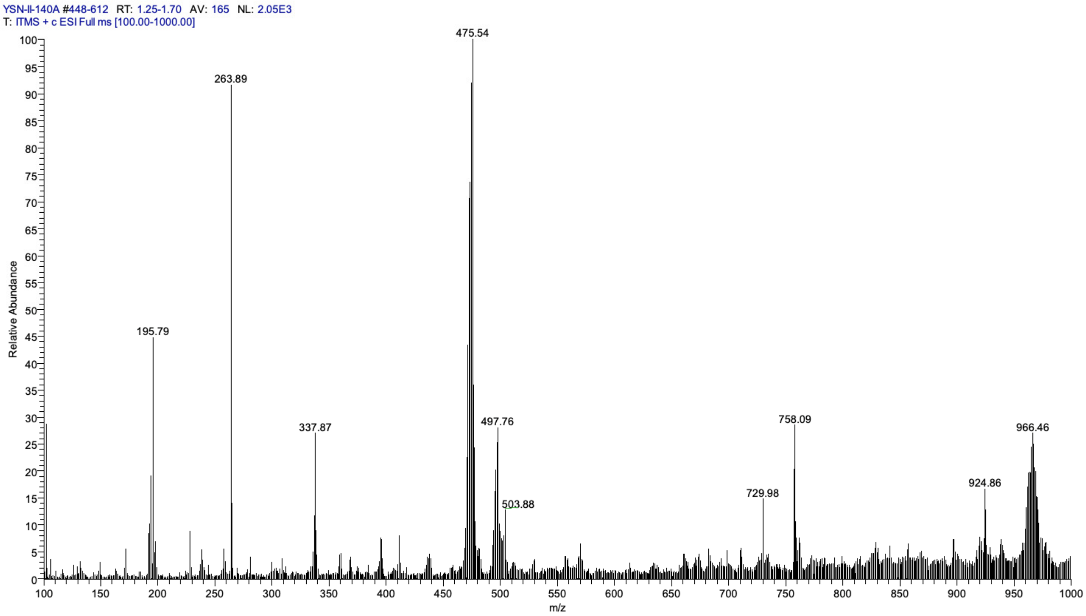
YSN-II-140A #448-612 RT: 1.25-1.70 AV: 165 NL: 2.05E3
T: ITMS + c ESI Full ms [100.00-1000.00]
475.54
263.89
195.79
337.87
497.76
503.88
758.09
729.98
966.46
924.86
Relative Abundance
m/z

5e MS

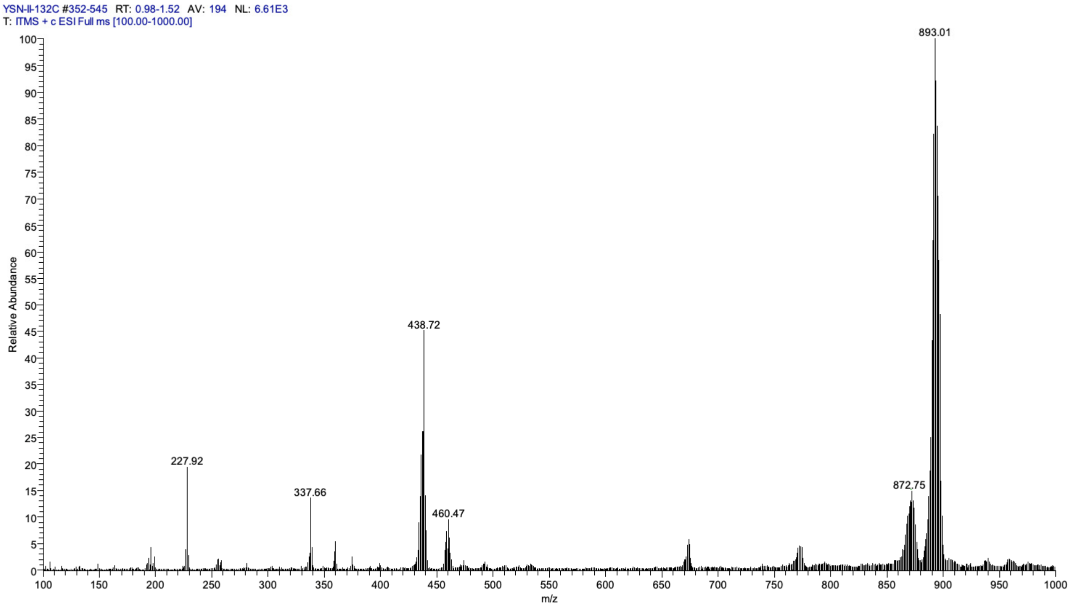
YSN-II-132C #352-545 RT: 0.98-1.52 AV: 194 NL: 6.61E3
T: ITMS + c ESI Full ms [100.00-1000.00]
893.01
438.72
227.92
337.66
460.47
872.75
Relative Abundance
m/z

5f MS

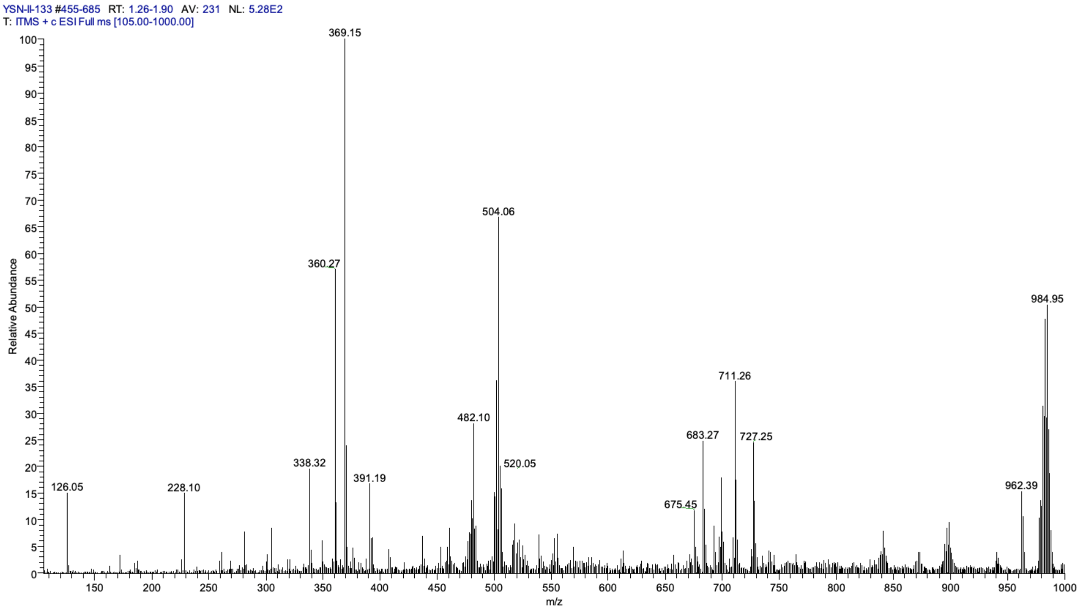
YSN-II-133 #455-685 RT: 1.26-1.90 AV: 231 NL: 5.28E2
T: ITMS + c ESI Full ms [105.00-1000.00]
369.15
504.06
360.27
984.95
711.26
482.10
683.27
727.25
338.32
520.05
126.05
228.10
391.19
962.39
675.45
Relative Abundance
m/z

5g MS

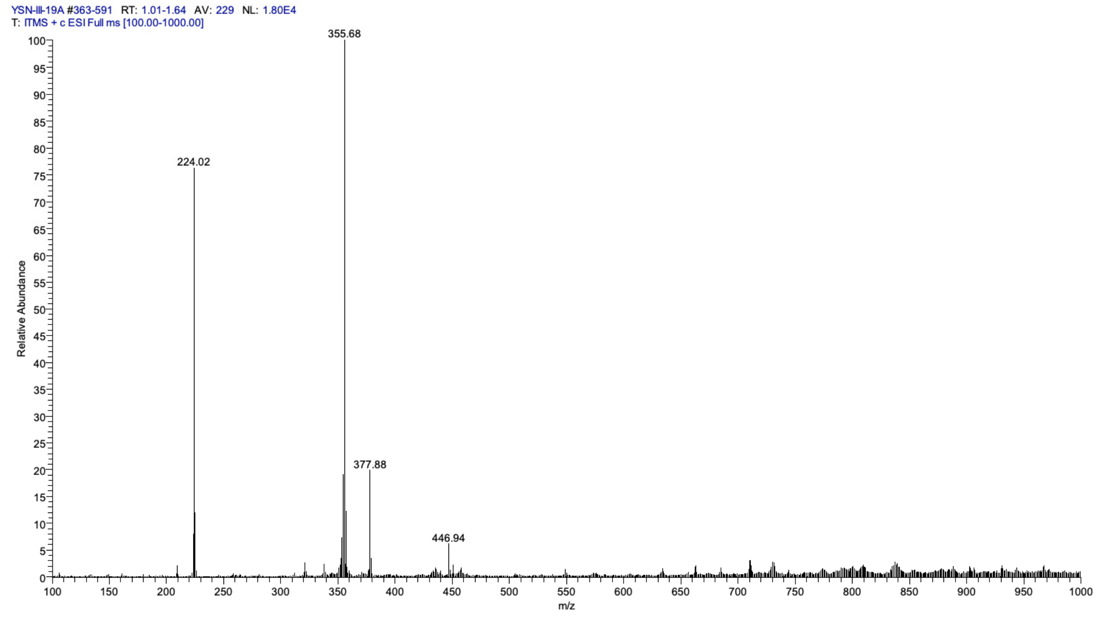
YSN-III-19A #363-591 RT: 1.01-1.64 AV: 229 NL: 1.80E4
T: ITMS + c ESI Full ms [100.00-1000.00]
355.68
224.02
377.88
446.94
Relative Abundance
m/z

5h MS

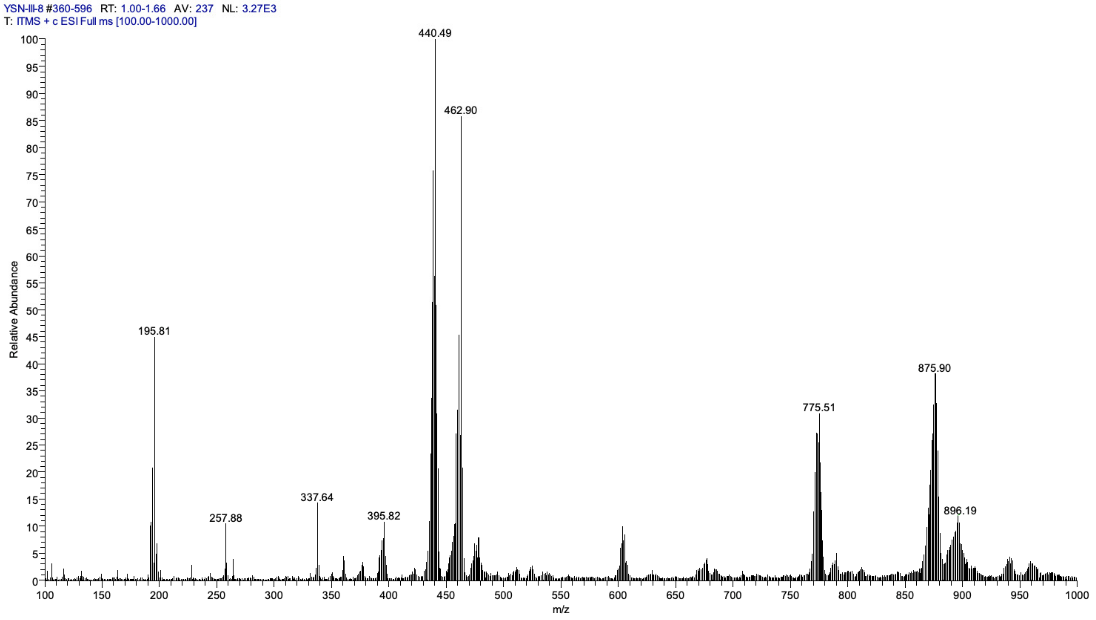
YSN-III-8 #360-596 RT: 1.00-1.66 AV: 237 NL: 3.27E3
T: ITMS + c ESI Full ms [100.00-1000.00]
440.49
462.90
195.81
257.88
337.64
395.82
775.51
875.90
896.19
Relative Abundance
m/z

6a MS

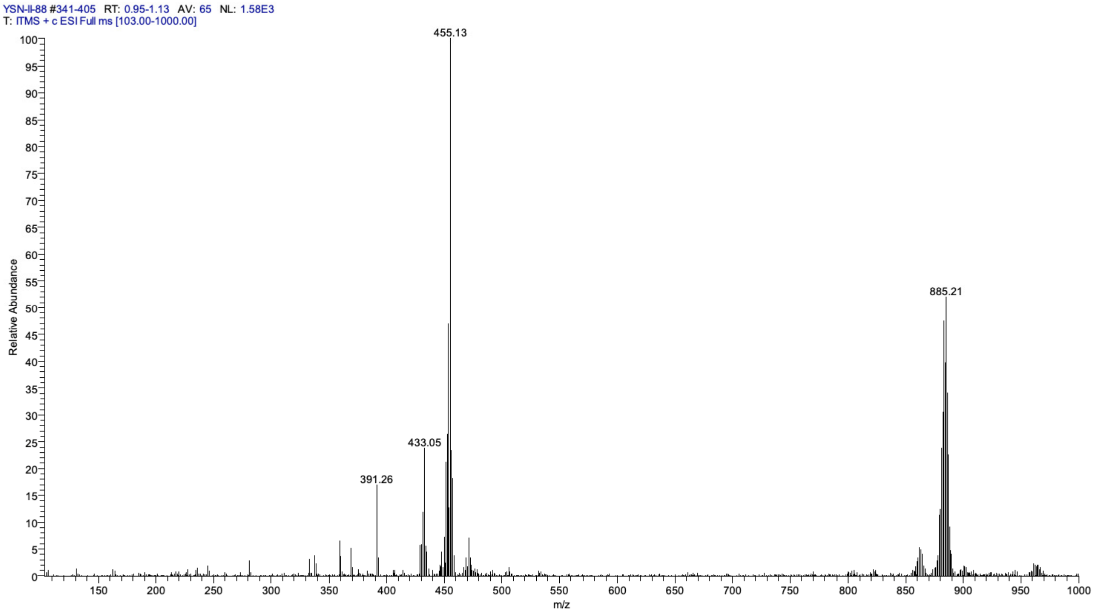
YSN-II-88 #341-405 RT: 0.95-1.13 AV: 65 NL: 1.58E3
T: ITMS + c ESI Full ms [103.00-1000.00]
455.13
885.21
433.05
391.26
Relative Abundance
m/z

6b MS

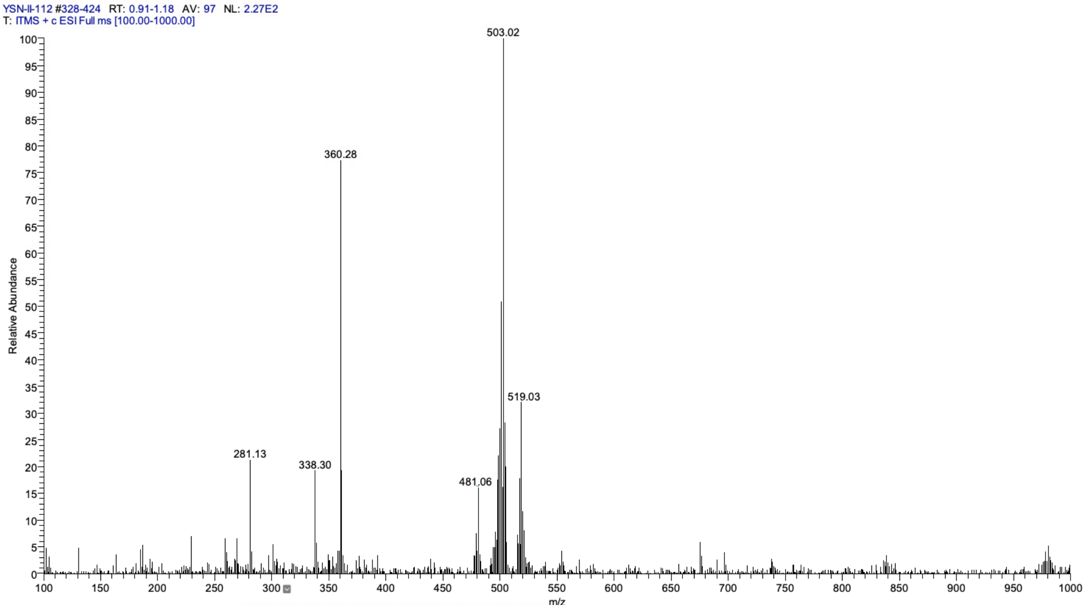
YSN-II-112 #328-424 RT: 0.91-1.18 AV: 97 NL: 2.27E2
T: ITMS + c ESI Full ms [100.00-1000.00]
503.02
360.28
519.03
281.13
338.30
481.06
Relative Abundance
m/z

6c MS

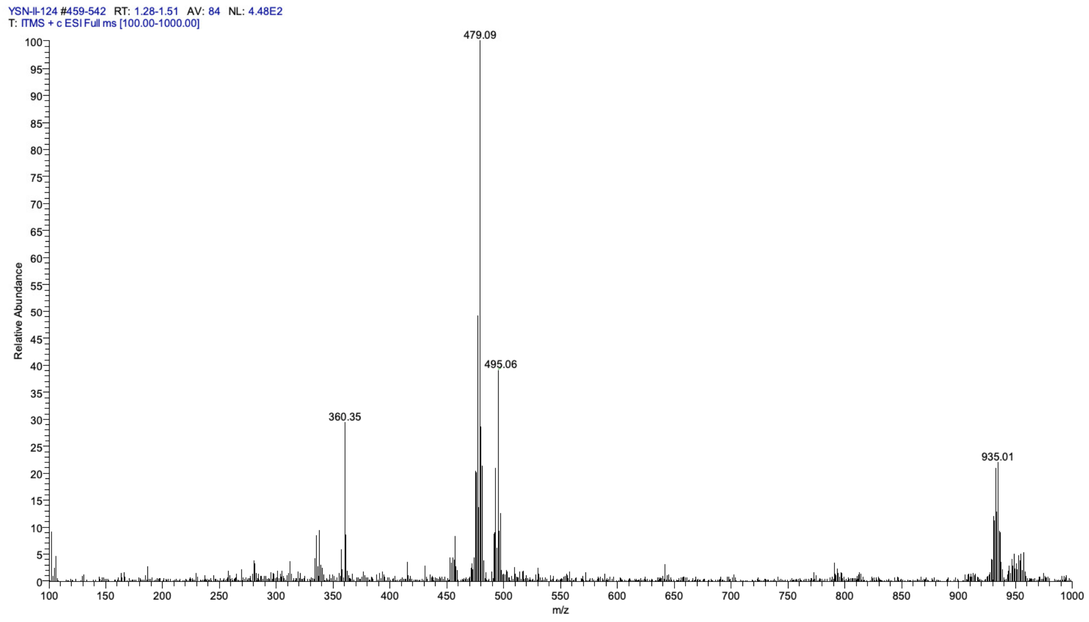
YSN-II-124 #459-542 RT: 1.28-1.51 AV: 84 NL: 4.48E2
T: ITMS + c ESI Full ms [100.00-1000.00]
479.09
495.06
360.35
935.01
Relative Abundance
m/z

6d

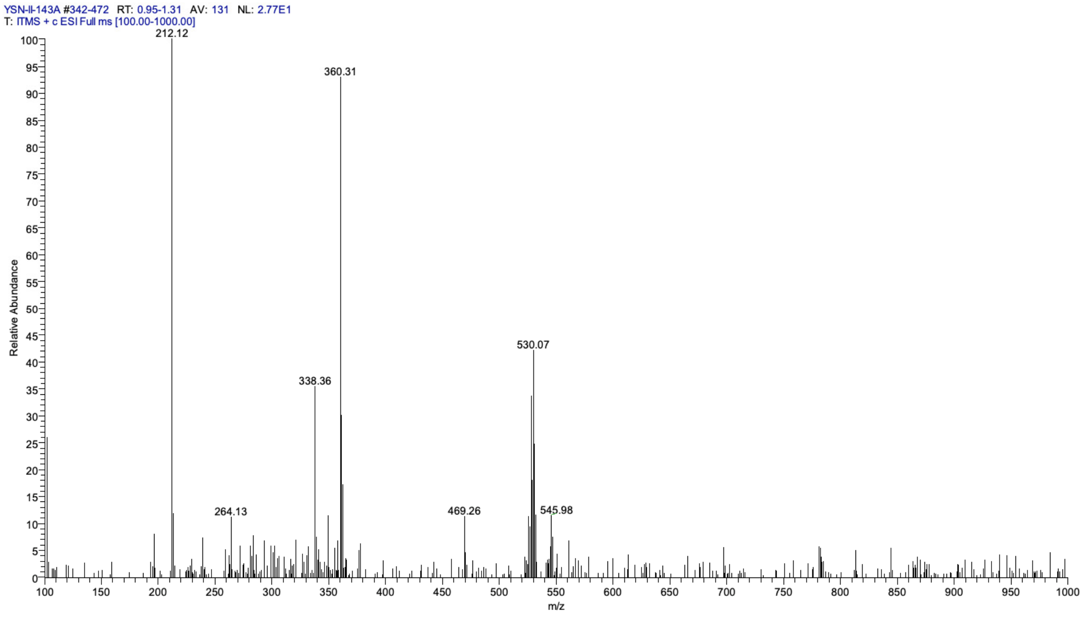
YSN-II-143A #342-472 RT: 0.95-1.31 AV: 131 NL: 2.77E1
T: ITMS + c ESI Full ms [100.00-1000.00]
212.12
360.31
530.07
338.36
264.13
469.26
545.98
Relative Abundance
m/z

6e MS

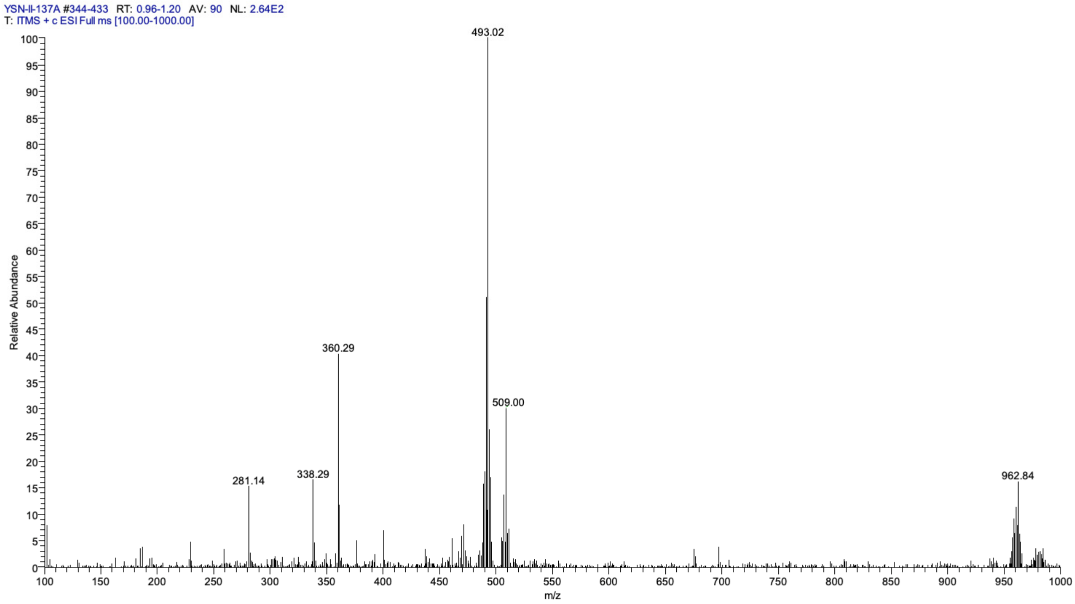
YSN-II-137A #344-433 RT: 0.96-1.20 AV: 90 NL: 2.64E2
T: ITMS + c ESI Full ms [100.00-1000.00]
Relative Abundance
m/z
493.02
360.29
509.00
281.14
338.29
962.84

6f MS

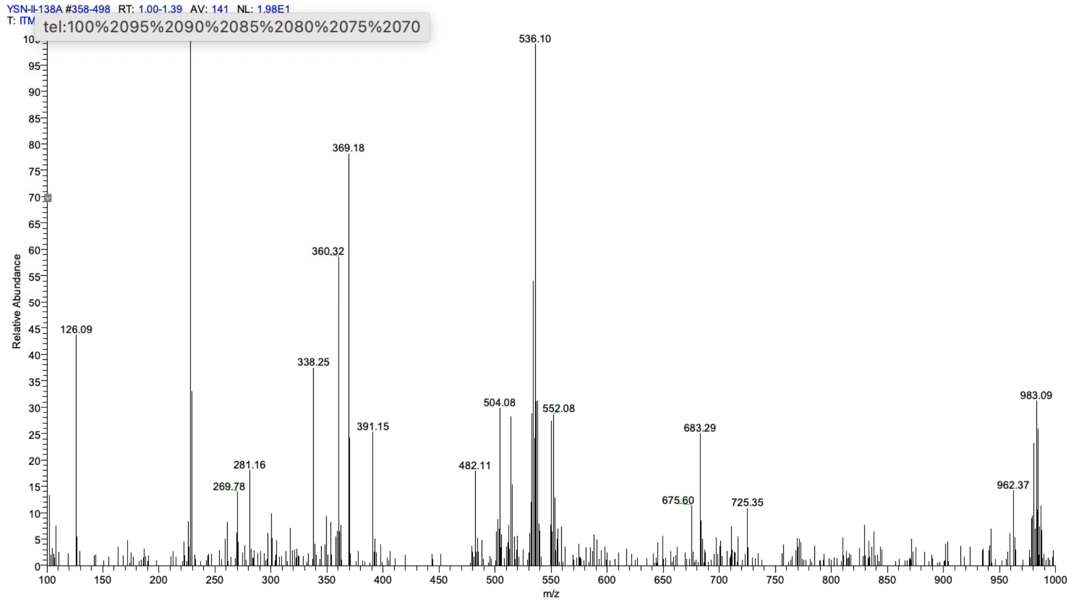
YSN-II-138A #358-498 RT: 1.00-1.39 AV: 141 NL: 1.98E1
tel:100%2095%2090%2085%2080%2075%2070
Relative Abundance
m/z
536.10
369.18
360.32
126.09
338.25
504.08
552.08
391.15
683.29
983.09
281.16
482.11
269.78
962.37
675.60
725.35

6g MS

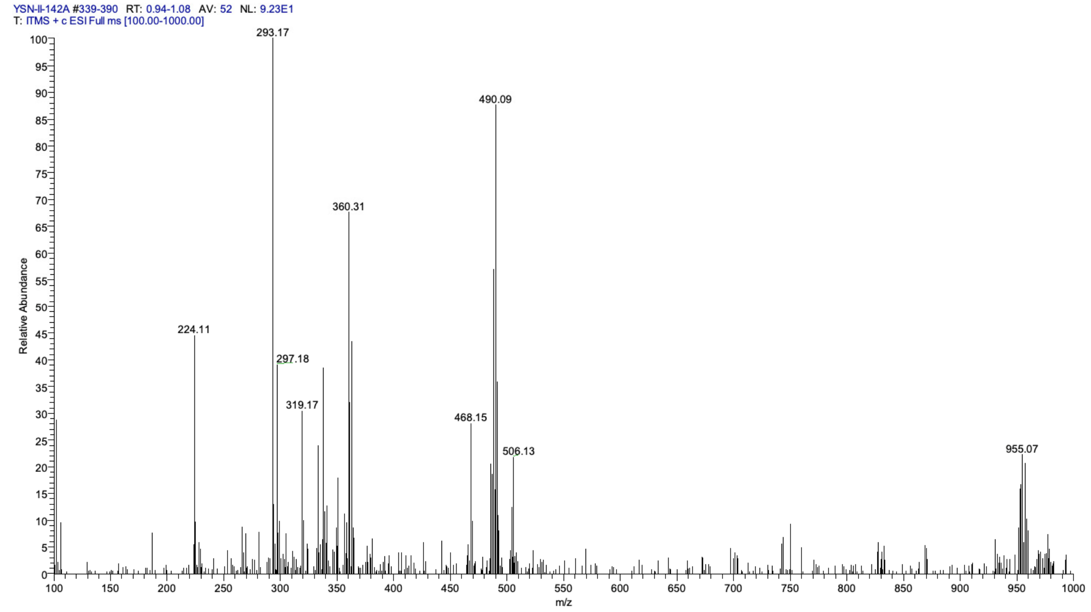
YSN-II-142A #339-390 RT: 0.94-1.08 AV: 52 NL: 9.23E1
T: ITMS + c ESI Full ms [100.00-1000.00]
Relative Abundance
m/z
293.17
490.09
360.31
224.11
297.18
319.17
468.15
506.13
955.07

6h MS

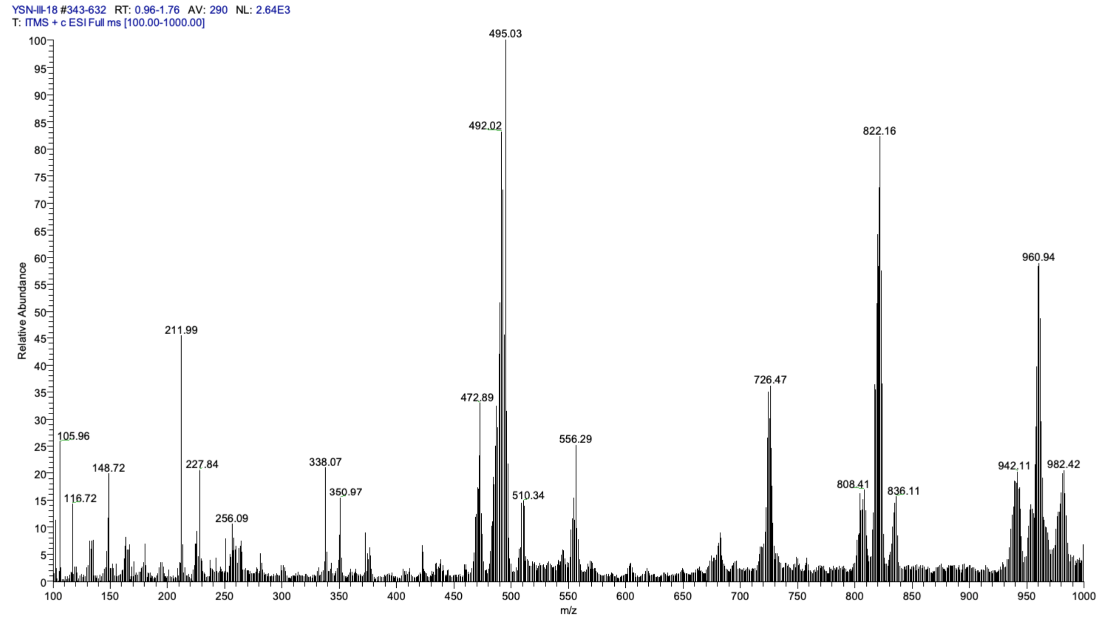
YSN-III-18 #343-632 RT: 0.96-1.76 AV: 290 NL: 2.64E3
T: ITMS + c ESI Full ms [100.00-1000.00]
Relative Abundance
m/z
495.03
492.02
822.16
960.94
211.99
726.47
472.89
105.96
556.29
148.72
227.84
338.07
808.41
942.11
982.42
116.72
350.97
510.34
836.11
256.09
